# Assessing generalisability of deep learning-based polyp detection and segmentation methods through a computer vision challenge

**DOI:** 10.1038/s41598-024-52063-x

**Published:** 2024-01-23

**Authors:** Sharib Ali, Noha Ghatwary, Debesh Jha, Ece Isik-Polat, Gorkem Polat, Chen Yang, Wuyang Li, Adrian Galdran, Miguel-Ángel González Ballester, Vajira Thambawita, Steven Hicks, Sahadev Poudel, Sang-Woong Lee, Ziyi Jin, Tianyuan Gan, ChengHui Yu, JiangPeng Yan, Doyeob Yeo, Hyunseok Lee, Nikhil Kumar Tomar, Mahmood Haithami, Amr Ahmed, Michael A. Riegler, Christian Daul, Pål Halvorsen, Jens Rittscher, Osama E. Salem, Dominique Lamarque, Renato Cannizzaro, Stefano Realdon, Thomas de Lange, James E. East

**Affiliations:** 1 School of Computing, Faculty of Engineering and Physical Sciences, University of Leeds, Leeds, LS2 9JT UK; 2 Department of Engineering Science, Institute of Biomedical Engineering, University of Oxford, Oxford, OX3 7DQ UK; 3 Oxford National Institute for Health Research Biomedical Research Centre, Oxford, OX4 2PG UK; 4 Computer Engineering Department, Arab Academy for Science and Technology, Smart Village, Giza, Egypt; 5 SimulaMet, 0167 Oslo, Norway; 6 Department of Computer Science, UiT The Arctic University of Norway, Hansine Hansens veg 18, 9019 Tromsø, Norway; 7 Graduate School of Informatics, Middle East Technical University, 06800 Ankara, Turkey; 8 City University of Hong Kong, Kowloon, Hong Kong; 9 BCN MedTech, Department of Information and Communication Technologies, Universitat Pompeu Fabra, 08018 Barcelona, Spain; 10 ICREA, Barcelona, Spain; 11 Department of IT Convergence Engineering, Gachon University, Seongnam, 13120 Republic of Korea; 12 College of Biomedical Engineering and Instrument Science, Zhejiang University, Hangzhou, 310027 China; 13 Tsinghua Shenzhen International Graduate School, Tsinghua University, Shenzhen, 518055 China; 14 Department of Automation, Tsinghua University, Beijing, 100084 China; 15 Smart Sensing and Diagnosis Research Division, Korea Atomic Energy Research Institute, Taejon, 34057 Republic of Korea; 16 Daegu-Gyeongbuk Medical Innovation Foundation, Medical Device Development Center, Taegu, 427724 Republic of Korea; 17 NepAL Applied Mathematics and Informatics Institute for Research (NAAMII), Kathmandu, Nepal; 18 Computer Science Department, University of Nottingham, Malaysia Campus, 43500 Semenyih, Malaysia; 19 Computer Science, Edge Hill University, Lancashire, United Kingdom; 20 CRAN UMR 7039, Université de Lorraine and CNRS, 54500 Vandœuvre-Lès-Nancy, France; 21 Oslo Metropolitan University, Pilestredet 46, 0167 Oslo, Norway; 22 Faculty of Medicine, University of Alexandria, Alexandria, 21131 Egypt; 23 Université de Versailles St-Quentin en Yvelines, Hôpital Ambroise Paré, 9 Av. Charles de Gaulle, 92100 Boulogne-Billancourt, France; 24 CRO Centro Riferimento Oncologico IRCCS Aviano Italy, Via Franco Gallini, 2, 33081 Aviano, PN Italy; 25 Veneto Institute of Oncology IOV-IRCCS, Via Gattamelata, 64, 35128 Padua, Italy; 26 Medical Department, Sahlgrenska University Hospital-Mölndal, Blå stråket 5, 413 45 Göteborg, Sweden; 27 Department of Molecular and Clinical Medicine, Sahlgrenska Academy, University of Gothenburg, 41345 Göteborg, Sweden; 28 Augere Medical, Nedre Vaskegang 6, Oslo, 0186 Norway; 29 Translational Gastroenterology Unit, Nuffield Department of Medicine, Experimental Medicine Division, John Radcliffe Hospital, University of Oxford, Oxford, OX3 9DU UK

**Keywords:** Biomedical engineering, Colonoscopy

## Abstract

Polyps are well-known cancer precursors identified by colonoscopy. However, variability in their size, appearance, and location makes the detection of polyps challenging. Moreover, colonoscopy surveillance and removal of polyps are highly operator-dependent procedures and occur in a highly complex organ topology. There exists a high missed detection rate and incomplete removal of colonic polyps. To assist in clinical procedures and reduce missed rates, automated methods for detecting and segmenting polyps using machine learning have been achieved in past years. However, the major drawback in most of these methods is their ability to generalise to out-of-sample unseen datasets from different centres, populations, modalities, and acquisition systems. To test this hypothesis rigorously, we, together with expert gastroenterologists, curated a multi-centre and multi-population dataset acquired from six different colonoscopy systems and challenged the computational expert teams to develop robust automated detection and segmentation methods in a crowd-sourcing Endoscopic computer vision challenge. This work put forward rigorous generalisability tests and assesses the usability of devised deep learning methods in dynamic and actual clinical colonoscopy procedures. We analyse the results of four top performing teams for the detection task and five top performing teams for the segmentation task. Our analyses demonstrate that the top-ranking teams concentrated mainly on accuracy over the real-time performance required for clinical applicability. We further dissect the devised methods and provide an experiment-based hypothesis that reveals the need for improved generalisability to tackle diversity present in multi-centre datasets and routine clinical procedures.

## Introduction

Colorectal cancer (CRC) is the third leading cause of cancer deaths, with a reported mortality rate of nearly 51%^1^. CRC can be characterised by early cancer precursors such as adenomas or serrated polyps that may, over time, lead to cancer. While polypectomy is a standard technique to remove polyps^2^ by placing a snare (thin wire loop) around the polyp and closing it to cut through the polyp tissue either with diathermy (heat to seal vessels) or without (cold snare polypectomy), identifying small or flat polyps (e.g. lesion less than 10 mm) can be extremely challenging. This is due to the complex organ topology of the colon and rectum that makes navigation and treatment procedures difficult and requires expert-level skills. Similarly, the localisation and removal of polyps can be very challenging due to constant organ deformations, making it sometimes impossible to keep track of the lesion boundary, making the complete resection difficult and subjective to the endoscopists’ experience. Computer-assisted systems can help to reduce operator subjectivity and improve adenoma detection rates (ADR). Similarly, computer-aided detection and segmentation methods can also assist in localising polyps and guiding surgical procedures (e.g. polypectomy) by showing the polyp locations and margins. Some of the major requirements of such a system to be utilised in the clinic are real-time performance and algorithmic robustness. The detection task involves both the classification and localisation of polyps, whereas segmentation provides the grouping of pixels in an image that are associated with an object belonging to the same category.

Machine learning advances, in particular deep learning, and tremendous improvements in hardware have enabled the possibility to design deeper neural networks that can provide real-time performance despite their complexity. However, one major challenge in developing these methods is the lack of comprehensive public datasets that include diverse patient populations, imaging modalities and endoscope manufacturers. Incorporating real-world challenges in the dataset can only be the way forward in building guaranteed robust systems. There have been several attempts to collect and curate gastrointestinal (GI) datasets that include other GI lesions and polyps (Supplementary Table 1). A significant limitation of the publicly available datasets is that they consist of a single centre or a data cohort representing a single population. The most widely used public datasets have sampled frames and consist of mostly single modality images. Moreover, even though conventional white-light endoscopy (WLE) is used in standard colonoscopic procedures, narrow-band imaging (NBI), a type of virtual chromo-endoscopy, is widely used by experts for polyp identification and characterisation.

For polyp, most deep learning-based detection^3–5^ and segmentation^6–9^ methods are trained and tested on the same centre dataset and WLE modality only. In the literature, there are two types of frameworks for object detection: single-stage detection framework and multi-stage detection framework. Segmentation deep learning methods can be generally classified into fully convolutional networks (FCN), Encoder–Decoder architecture, pyramid-based and dilate convolution-based architectures^10^. All of these method types have been explored by different groups in their works localisation and segmentation tasks of polyps. Details on the methodologies for both of these tasks for polyp can be found in the “Related work” section of the Supplementary Notes. It is important to note that most of these methods are supervised deep learning techniques that have a major issue in not being able to generalise to unseen data from a different centre population^11^ or even different modality from the same centre^12^. The type of endoscope used also adds to the compromise in robustness. Due to selective image samples provided by most of the available datasets for method development, the test dataset is also comprised of similarly collected set data samples^9,13,14^. Like most endoscopic procedures, colonoscopy is a continuous visualisation of mucosa with a camera and a light source. During this process, live videos are acquired, which are often corrupted with specularity, floating objects, stool, bubbles and pixel saturation^15^. The mucosal scene dynamics such as severe deformations, view-point changes, and occlusion can be major limiting factors for algorithm performance. It is thus important to cross-examine the generalisability of developed algorithms more comprehensively and on variable data settings, including modality changes and continuous frame sequences. These challenges often lead to the failure of medical image analysis methods. Even for the current CNN-based methods imaging artefacts can cause either no detection of polyps or poor accuracies. Similarly, for segmentation methods where precise boundary recognition is important, these challenges often tend algorithms to under or over-segment areas that can affect automated therapy or resection procedures leading to sub-optimal treatment causing the re-occurrence of polyps.

With the presented crowd-sourced Endoscopic Computer Vision challenge in 2021 (EndoCV2021) conducted in conjunction with the IEEE International Symposium on Biomedical Imaging (ISBI), we collected and curated a multicentre dataset^16^ aiming at the generalisability assessment of colonoscopy polyp detection and polyp segmentation challenge tasks. For this, we took a strategic approach of providing single modality (white light endoscopy modality, WLE) data from five hospitals (both single frame and sequence) for training and validation while the test data consisted of four different real-world colonoscopy configurations—(a) mixed centre unseen data with WLE modality comprising of samples from five centres also present in the training data, (b) a different modality data (narrow-band imaging modality, NBI) from all centres for testing only, (c) a hidden sixth centre single frame data for testing only and (d) a hidden sixth centre continuous frame sequence data for testing only. While hold-out data with centres included in training assesses the traditional way of testing the supervised machine learning methods on held-out data, unseen modality and hidden centre test data gauge the algorithm’s generalisability. Similarly, sequence test data split mimics the occurrence of polyps in data as observed in routine clinical colonoscopy procedures. The same data was used for assessing both detection and segmentation tasks.

**Figure 1:**
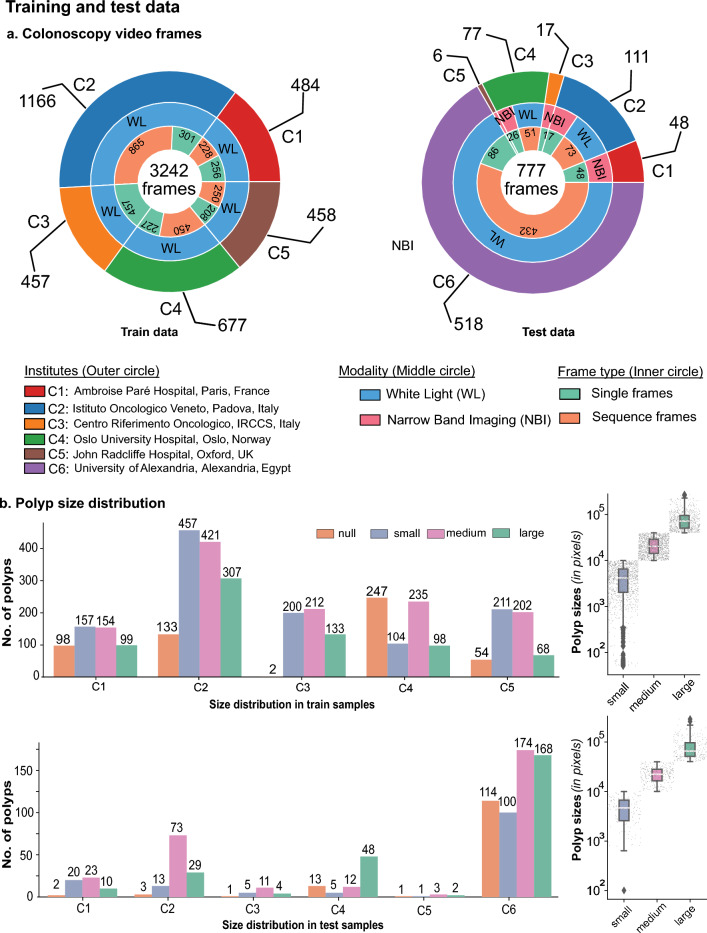
Multi-centre training and test samples. (**a**) Colonoscopy video frames for which the annotation samples were reviewed and released as training (left) and test (right) are provided. Training samples included nearly proportional frames from five centres (C1–C5). In contrast, test samples consisted of a majority of single and sequence frames from the unseen centre (C6) with white light modality (WL) only. Test data from the seen centres C1, C3, and C5 consisted of only NBI images, while centres C2 and C4 consisted of white light (WL) and narrow-band imaging (NBI) modalities. (**b**) The number of polyp counts and samples with no polyps per centre are provided. Polyp sizes (in pixels) were classified based on resized image frames of $540\times 720$ pixels. Polyp sizes (in pixels) are provided on the left, along with their intra-size variability (in log10 scale) on the right for training (top) and testing data (bottom).

**Figure 2:**
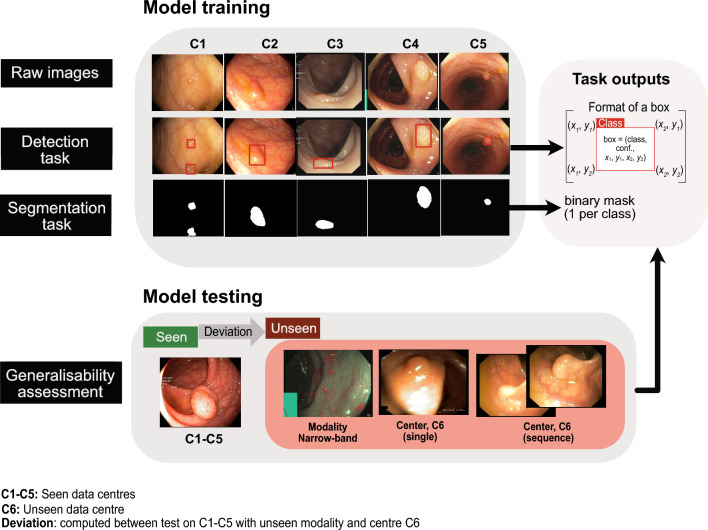
EndoCV2021 challenge tasks: participants performed model training on white light imaging data collected from five centres (C1–C5). The tasks included detection and segmentation. Trained models were then tested on both seen and unseen centre datasets and on unseen data modality (widely used narrow-band imaging). Generalisability assessment is obtained by computing deviations between these unseen samples w.r.t. seen samples. Task outputs included bounding box prediction with confidence and class label for detection task and binary mask prediction for polyp segmentation.

## Material and methods

### Dataset

The EndoCV2021 challenge addresses the generalisability of polyp detection and segmentation tasks in endoscopy frames. The colonoscopy video frames utilised in the challenge are collected from six different centres, including two modalities (i.e. WL and NBI) with both sequence and non-sequence frames (Fig. 1a). The challenge included five different types of data: (1) multi-centre video frames from five centres for training and validation, (2) polyp size-based, (3) single frame and sequence data split, (4) modality split (only for testing phase), and (5) one hidden centre test (test phase only). The training dataset consisted of 3242 WL frames from five centres (i.e. C1–C5) with both single and sequence frames. Participants were allowed to combine accordingly for their train-validation splits. The test dataset consists of (a) dataset with unseen modality, NBI (data 1), (b) dataset with single frames from the unknown centre (data 2), (c) frame sequences from the mixed centres (C1–C5, data 3), and iv) the unseen centre sequence frames (C6, data 4). A total of 777 frames were used, and data 3 was picked as the base dataset against which the generalisability of methods were assessed. Polyp size distribution (Fig. 1b, on left) and its size in log-scale on resized images of the same resolution ($540\times 720$ pixels) (see Fig.  1b, right) in both training and test sets are presented. These sizes were divided into null (for no polyp in frames), small ($< 100 \times 100$ pixels bounding box), medium (between $100 \times 100$ pixels and $200\times 200$ pixels polyp bounding box) and large ($> 200\times 200$ pixels polyp bounding box). These numbers were 534, 1129, 1224 and 705, respectively, for null, small, medium and large polyps (accounting for 3058 polyp instances) in the training set. Similarly, for the test set, the numbers were 134, 144, 296 and 261, respectively, for null, small, medium and large size polyps (in total 701 polyp instances). The size variation in both datasets is nearly identical i.e., there are similar variations in different polyp sizes (in pixels) (Fig. 1b, on the right), which is due to the defined range for categorically representing their occurrence.

### Annotation protocol

The annotation process was conducted by a team of three experienced researchers using an online annotation tool called Labelbox (see https://labelbox.com). Each annotation was cross-validated by the team and by the centre expert for accurate polyp boundaries segmentation. At least one senior gastroenterologist was assigned for an independent binary review process. A set of protocols for manual annotation of polyp were designed as follows:

- Clear raised polyps: Boundary pixels should include only protruded regions. Precautions were taken when delineating along the normal colon folds.
- Inked polyp regions: Only part of the non-inked appearing object delineation
- Polyps with instrument parts: Annotation should not include instrument and is required to be carefully delineated and may form more than one object
- Pedunculated polyps: Annotation should include all raised regions unless appearing on the fold
- Flat polyps: Zooming the regions identified with flat polyps before manual delineation. Also, consulting a centre expert if needed.

The annotated masks were examined by experienced gastroenterologists who gave a binary score indicating whether a current annotation can be considered clinically acceptable or not. Additionally, some experts provided feedback on the annotation where these images were placed into an ambiguous category for further refinement based on the expert’s feedback. A detailed process along with the number of annotations conducted and reviewed is outlined in Supplementary Fig. 1, and a few exemplary labels for each protocol case are shown in Supplementary Figure 2.

### Challenge tasks:

EndoCV2021 included two tasks (see Fig. 2): (1) detection and localisation task and (2) pixel-level segmentation task. For both the tasks generalisability assessment was also conducted. For the detection task, participants were provided with single and sequence frames with manually annotated ground truth polyp labels and their corresponding bounding box locations (origin, height, and width). Participants were required to train their model for predicting “polyp” class label, bounding box coordinates (origin, height, and width), and confidence scores for localisation. For the semantic segmentation task, the pixel-level segmentation ground truths from experts were provided that included the same data as provided for the detection task. The participants were challenged to obtain close-to-ground truth binary map prediction for each pixel (zero for background and 1 for polyp). Both of these challenge tasks were assessed rigorously to understand the generalisability of the developed methods. In this regard, the test data consisted of four different categories: data 1, data 2, data 3 and data 4. Data 1 consisted of unseen modality with NBI data widely used in colonoscopy; data 2 comprised single frames of unseen centre C6; data 3 consisted of mixed seen centre (C1–C5) sequence data. In contrast, data 4 included sequence data from unseen centre C6. The scores between data 3 (seen centre data) were compared with the other unseen data categories for generalisability assessment. All test results were evaluated on a standard NVIDIA Tesla V100 GPU. Tabulated summaries are provided highlighting the nature of the devised methods and basis of choice in terms of speed and accuracy for detection and segmentation (see Table 1). Most of the participating teams were motivated on building ensemble models to benefit from the advantages provided by the ensemble concept. The process of ensemble learning incorporates several unique models to achieve better generalisation performance, reduce generalisation error and provide improved predictive accuracy when compared to the individual models^17^.

### Method summary for participating teams

In this paper, we have dissected only the top 4 teams for detection and the top 5 for segmentation based on the final leaderboard for round two of the challenge (https://endocv2021.grand-challenge.org/evaluation/round-ii-segmentation-genralization-challenge/leaderboard/). For the detection tasks, team AIM_CityU^18^ employed the one-stage anchor-free FCOS as the core detection algorithm and chose ResNeXt-101-DCN with FPN as their final feature extractor. Team JIN_ZJU^19^ proposed several data augmentation techniques to train the standard YOLOV5 as the baseline detection algorithm. An ensemble-based architecture for polyp detection was developed, utilizing the EfficientDet model by team GECE_VISION^20^ by aggregating various versions of the Efficient predictors.

For the segmentation task, team aggcmab^21^ proposed approach involves employing a cascaded double encoder–decoder convolutional neural network. This network architecture aims to enhance the representation capabilities of the encoder while also adjusting to a multi-site selection method. Team AIM_CityU^18^ has put out a suggestion for a low-rank module that aims to distribute feature mappings from a high-dimensional space to a low-dimensional manifold while using HRNet as the backbone network. Furthermore, team MLC_SimulaMet^22^ proposed a two-ensemble modelthat incorporates several segmentation methods and a new TriUNet for their DivergentNet ensemble model. Additionally, team sruniga^23^ also used the HarDNet-MSEG as the backbone network and made sure there were few shortcuts. They also used a data augmentation strategy to make the model more general. Finally, team HoLLYS_ETRI^24^ utilized the Mask R-CNN framework in *both* task detection and segmentation of the polyps. Ensemble learning was employed, using a 5-fold cross-validation approach to improve the overall performance.

**Figure 3:**
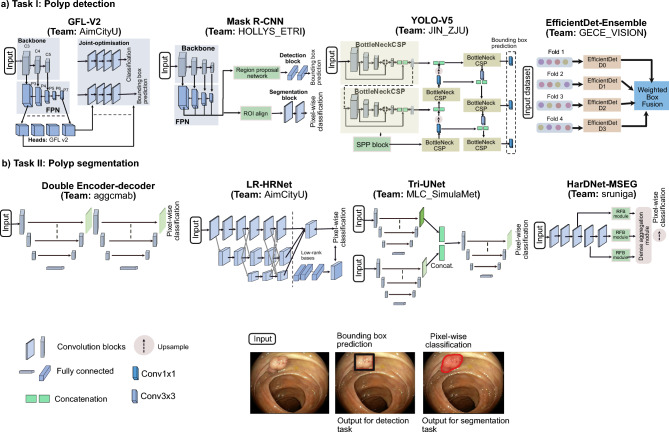
Deep learning methods for segmentation and detection of colonoscopy polyps: method design of all top teams for detection and segmentation tasks are depicted. Each network is fed with an input image and the output prediction is then either directly predicted or an ensemble of network is used for prediction. The description of backbone and nature of these networks is provided in Table 1. Each output prediction for detection task is bounding box prediction with class label polyp while for segmentation are the pixel-wise classification where polyp classes are provided with label 1 and background pixels as label 0. At bottom, an overlay on the original image, bounding box prediction (in black) and segmentation prediction (in red) are also shown.

Main network framework devised by five top teams are illustrated in Fig. 3. More details about top-performing teams participating in detection and segmentation tasks are in a later section.

### Evaluation metrics

Assessment of challenge tasks was conducted both on widely used standard metrics and novel generalisation metrics developed by the organisers to determine performance gaps between different test-splits (for reproducibility see https://github.com/sharib-vision/EndoCV2021-polyp_det_seg_gen).

#### Polyp detection

For the polyp detection task, standard computer vision metrics such as average precision (AP) and intersection-of-union (IoU)^25^ defined below were computed.

- IoU: The IoU metrics measures the overlap between two bounding boxes A and B as the ratio between the target mask and predicted output, $\text {IoU(A,B)} =\frac{A \cap B}{A \cup B}$. Here, $\cap$ represents intersection and $\cup$ represents the union.
- AP: AP is computed as the area under curve (AUC) of the precision-recall curve of detection sampled at all unique recall values ($r1, r2,\ldots$) whenever the maximum precision value drops. The mathematical formulation is given by: $\textrm{AP} = \sum _n{\left\{ \left( r_{n+1}-r_{n}\right) p_{\textrm{interp}}(r_{n+1})\right\} }$. Here, $p_{\textrm{interp}}(r_{n+1}) =\underset{\tilde{r}\ge r_{n+1}}{\max }p(\tilde{r})$. Here, $p(r_n)$ denotes the precision value at a given recall value. This definition ensures monotonically decreasing precision. AP was computed as an average APs at 0.50 and 0.95 with the increment of 0.05. Additionally, we have calculated AP_small_, AP_medium_, AP_large_. More description about the detection evaluation metrics and their formulas are provided at this link (see:  https://github.com/sharib-vision/EndoCV2021-polyp_det_seg_gen/blob/main/evaluationMetrics.

#### Polyp segmentation

For polyp segmentation task, widely accepted computer vision metrics were used that include Sørensen-Dice Coefficient ($DSC = \frac{2 \cdot tp}{2 \cdot tp + fp + fn}$), Jaccard Coefficient ($JC= \frac{tp}{tp + fp + fn}$), precision ($p=\frac{tp}{tp + fp}$), recall ($r= \frac{tp}{tp + fn}$), overall accuracy ($Acc = {\frac{tp + tn}{tp + tn + fp + fn}}$ ), and F2 ($=\frac{5p \times r}{4p + r}$). Here, *tp*, *fp*, *tn*, and fn represent true positives, false positives, true negatives, and false negatives, respectively. In addition to the performance metrics, run time of each algorithm was also computed and reported in milliseconds ms.

Another commonly used segmentation metric that is based on the distance between two point sets, ground truth (G) and estimated or predicted (E) pixels, was used. The metric is known as an average Hausdorff distance ($H_{d}$) and formulated as $H_{d}(G, E) = \bigg (\frac{1}{G} \sum _{g\in G} \min _{e\in E} d(g, e) + \frac{1}{E} \sum _{e\in E} \min _{g\in G} d (g, e)\bigg )/2$. $H_{d}$ is normalised between 0 and 1 by dividing it by the maximum value for a given test set. Thus, $1 - H_{d}$ can be considered as the range for which higher values represented smaller distance between ground truth and estimated segmentation boundaries.

#### Polyp generalisation metrics

Generalisability score was defined based on the stability of the algorithm performance on seen centre dataset with WLE modality (data 3) versus unseen centre splits (data 2 and data 4) and unseen modality (data 1) in the test dataset. We conducted the generalisability assessment for both detection and segmentation approaches separately.

For detection, the deviation in score between seen and unseen data types were computed over different *AP* categories, $k \in \{mean, small, medium, large\}$ with empirically set tolerance of 10% tolerance, ($tl = 0.1$):

1 $$\begin{aligned} dev\_g = \frac{1}{|k|}\sum _k{\left\{ \begin{array}{ll} |\mathrm {AP_k}^{seen} - \mathrm {AP_k}^{unseen}|, \text {if } \mathrm {AP_k}^{unseen} \ge \mathrm {AP_k}^{seen} - tl *\mathrm {AP_k}^{seen} \text { or } \mathrm {AP_k}^{unseen} \le \mathrm {AP_k}^{seen} + tl *\mathrm {AP_k}^{seen}\\ 0, \quad \text {otherwise.} \\ \end{array}\right. } \end{aligned}$$

Similarly, for segmentation, the deviation in score between seen and unseen data types are computed over different segmentation metric categories, $k \in \{DSC, F2, p, r, H_d\}$ with empirically set tolerance of 5%, ($tl = 0.05$):

2 $$\begin{aligned} dev\_g = \frac{1}{|k|}\sum _k{\left\{ \begin{array}{ll} |\mathrm {S_k}^{seen} - \mathrm {S_k}^{unseen}|, &{} \text {for } \mathrm {S_k}^{unseen} \ge \mathrm {S_k}^{seen} - tl *\mathrm {S_k}^{seen} \text { or } \mathrm {S_k}^{unseen} \le \mathrm {S_k}^{seen} + tl *\mathrm {S_k}^{seen}\\ 0, \quad \text {otherwise.} \\ \end{array}\right. } \end{aligned}$$

A higher tolerance value is chosen for detection compared to segmentation because the mean intersection-over-union overlap between the detected and ground truth boxes can have a 10% offset but can still localise the polyps well enough, while for segmentation, a larger change can refer to under or over-segmentation.

### Challenge setup, and ranking procedure

A challenge website with an automated docker system for metric-based ranking procedures was setup (see https://endocv2021.grand-challenge.org). Challenge participants were required to perform inference on our cloud-based system that incorporated NVIDIA Tesla V100 GPU and provided a test dataset with instructions for using GPU directly without downloading the data for the first two rounds. However, the round 3 was added to assess participant’s trained model on an additional unseen sequence dataset by the organisers and dissect the methods for fairness reporting and experiment-based hypothesis as learning lessons. Thus, the challenge consisted of three rounds, where all provided test frames were from unseen patient data to prevent data leakage. Further details on data samples in each round are summarised below:

- Round 1: This round consisted of a subset of test samples released in round 2 and 3. This test subset consisted of three data splits each with 50 image samples (in total 150 samples) including unseen modality (data 1, 50/135 samples), unseen single sample (data 2, C6, 50/86 samples) and mixed centre C1–C5 sequence data (data 3, 50/124 samples).
- Round 2: Test subset-II comprised 88 (out of 135) samples of data 1 (unseen modality), 86 samples of data 2 (unseen single sample, C6) and 124 samples of data 3 (mixed C1–C5). The total test subset-II comprised of 298 frames.
- Round 3: The organisers performed inference on round 3 data using the same GPU. This round comprised of a full test set with 135 samples of data 1 (unseen modality), 86 samples of data 2 (unseen single sample, C6), 124 samples of data 3 (mixed centre C1–C5 sequence data) and an additional set of 432 sequence samples (data 4) from unseen centre C6. Test data 4 was not used in rounds 1 and 2. The total test set thus comprised a total of 777 frames.

We conducted elimination for both round 1 and round 2 based on the metric scores on the leaderboard and timely submission. In round 2, we eliminated teams with very high computational time for inference (over two seconds) and low metric scores on the leaderboard. The metric criteria set for elimination for both rounds was 10% lower values compared to our baseline model evaluation on the results for worst performing test data (e.g., 0.10 on average AP and less than 0.50 on DSC). The chosen participants were requested for the method description paper at the EndoCV proceeding^26^ to allow transparent reporting of their methods. All accepted methods were eligible for round 3 evaluation and have been reported in this paper. Based on leaderboard valid submission, only eight top performing teams out-of 16 for segmentation task and four top performing teams teams out-of 6 teams for detection task were invited for round 2 and round 3 evaluations. Teams with lower scores and high processing time were also eliminated.

We rank teams in each category first. The categories for detection included—average detection scores across all data, deviation of each method with the training distribution (that is, held-out test data 3) and other distributions (test data 1, data 2 and data 4), and finally, the time. We then take the average of these rankings (the lowest ranking to be the best) and round them. Teams with the lowest value are sorted in ascending order to get the ranks. Similarly, for team ranking on the segmentation task, we used average segmentation score and deviation scores (between seen and unseen data) using the same datasets as used in the detection task. Each score including the time, were ranked individually first, and rounded. Teams with the lowest value are sorted in ascending order to get the ranks. All scores involved in the ranking were equally weighted. Please refer to section “Evaluation metrics”**** for details.

**Table 1:** Summary of the participating teams detection and segmentation tasks for the crowd-sourced polyp generalisation challenge.

Team name	Algorithm	Backbone	Nature	Choice basis	Data Aug.	Loss	Opt.	Code	No. of parameters (M)
Task I: polyp detection									
AIM_CityU^18^	FCOS	FPN, ResNeXt -101-DCN	ATSS	Accuracy speed	No	Generalized Focal loss	SGD	[d1]	51.0
HoLLYS_ETRI^24^	Mask R-CNN	FPN ResNet34	Ensemble	Accuracy++	No	Smooth L1	SGD	[d2]	63.75
JIN_ZJU^19^	YOLOV5	CSPdarknet SPP	Ensemble	speed++	Yes	BECLogits	SGD	[d3]	140.70
GECE_VISION^20^	EfficientDet	EfficientNet D0-D3	Ensemble	Accuracy	Yes	Focal loss	Adam	[d4]	30.60
Task II: Polyp segmentation									
Aggcmab^21^	DPN92-FPN	DPN92-FPN	Cascaded	Accuracy++	Yes	BCE	SGD	[s1]	75.91
AIM_CityU^18^	HRNet + LRM	HRNet	MSFF	Accuracy speed	Yes	BCE, DSC	SGD	[s2]	49.90
HoLLYS_ETRI^24^	Mask R-CNN	ResNet50	Ensemble	Accuracy+ speed+	Yes	Smooth L1	SGD	[s3]	63.75
MLC_SimulaMet^22^	DivergentNet	TriUNet	Ensemble	Accuracy++	No	BCE, DSC	Adam	[s4]	180.64
Sruniga^23^	HarDNet68	HarDNet68	Multiscale	Accuracy+ speed++	No	BCE	Adam	[s5]	17.42

All test was done on NVIDIA V100 GPU provided by the organisers. In total 11 different methods are provided together with the nature of these methods and basis of their choice that the teams considered. All codes for each team are available for reproducibility.

*FCOS* fully convolutional one-stage object detection, *FPN* feature pyramid network, *ATSS* adaptive training sample selection.

*YOLO* You Only Look Once, *SGD* Stochastic Gradient ‘escent, *[d1]–[d4]* hyperlinked GitHub repos.

*LRM* low-rank module, *MSFF* multi-scale feature fusion, *DPN* dual path network, *FPN* feature pyramid network, *BCE* binary cross entropy

*BCE* binary cross entropy, *DSC* dice similarity coefficient, *IoU* intersection over union, *W* weighted, *SGD* Stochastic gradient descent.

### Ethical approval and privacy aspects of the data

The EndoCV2021 data was gathered from 6 different centres in five countries (i.e. UK, Italy, France, Norway and Egypt). Each responsible centre handled the relevant data’s ethical, legal, and privacy (see Supplementary Table 2). All data used in the experiments were collected through informed patient consent. All institutions required institutional approvals. Collected imaging data were approved by IDRCB for the Ambroise Paré Hospital (Paris, France) (IDRCB: 2019-A01602-55); for John Radcliffe Hospital (Oxford, UK) institutional research ethics committee approved the collection and use of the data under REC Ref: 16/YH/0247, and other collected images at other centres were approved by the institutional data inspectorate. It is to be noted that no tissue samples were used. All imaging data used in this study were collected and fully anonymised following the General Data Protection Regulation (GDPR) and the Declaration of Helsinki. All methods were carried out in accordance with relevant guidelines and regulations.

## Results

Our crowd-sourced challenge focuses establishing an objective generalisation study for detection and segmentation of polyps. We collated a multi-centre dataset with 600 patients videos and consisted of polyps with variable sizes, presence of both single and sequence frames, an unseen modality and an unseen centre. The endoscopy video frames were gathered from six centres across varied populations (France, UK, Italy, Norway and Egypt), including two modalities (i.e. white light and narrow-band imaging). Annotations were made by three researchers and reviewed by expert GI consultants in the challenge. The training dataset consisted of 3242 colonoscopy video frames from five centres with both binary masks for the segmentation task and bounding box coordinates for the detection task. For the test dataset, frames from additional sixth centre were included to provide 777 frames from all six centres with a variation between single and sequence frames (see Fig. 1). There was a variation in the polyp sizes (in pixels) in both the training and testing set as shown in Fig. 1b. All image files provided in the training data were explicitly separated at both patient and video levels from the test samples to make sure that there is no data leakage.

**Figure 4:**
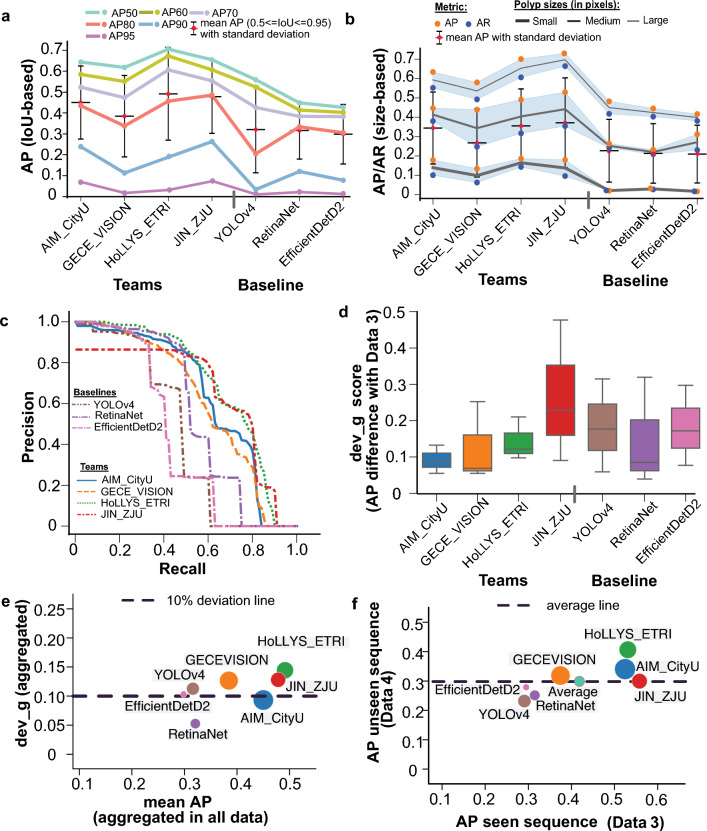
Assessment of detection methods: (**a**,**b**) demonstrate mean average precision (AP) in both IoU-based and polyp size-based average precision. The downward trend towards the right signifies lower values for the baselines than the team values. In (**b**), the top of the blueish region represents the average precision (AP), while the bottom represents the average recall (AR). The black line demonstrates the mean of these two values. Both AP and AR are preferred to be higher. (**c**) Precision-recall (PR) curve for detection task for all test sets (aggregated). A separate PR-curve for each test set is shown in Supplementary Figure 2. (**d**) Deviation in mAP scores for data 1, data 2 and data 4 *wrt* data 3. The box plot with a lower interquartile range and median value demonstrates lower deviation and improved generalisability. Clearly, most teams have lower deviations compared to baseline methods. (**e**,**f**) Generalisation assessment on detection task for which mean average precision (mAP) on all data versus deviation computed between seen centre with unseen modality and unseen centre is provided in (**e**). The least deviation (below the dashed line) with a larger mean average precision (mAP in the X-axis) is desired. Similarly, a comparison of mAP for both teams and baseline methods on seen centre sequence data (C1–C5, data 3) versus unseen centre sequence data, C6 (data 4). Higher values along both axes are desired. The size of the circle only refers to a different team or baseline method for better illustration.

### Aggregated performance and ranking on detection task

The average precision (AP) at multiple IoU thresholds and polyp-sizes were calculated to understand the preciseness in localisation of the detected polyps in the entire test dataset (Fig. 4a,b). Higher IoU thresholds mean better polyp localisation. It can be observed that HoLLYS_ETRI and JIN_ZJU teams provided the best two results for most IoU thresholds except for AP90 (i.e. IoU threshold of 0.90), where team AIM_CityU showed the second-best result (Fig. 4a). Similar observations can be noted for polyp size-based AP and average recall (AR), for which AIM_CityU has very close results to team JIN_ZJU for small polyps and to team HoLLYS_ETRI for medium polyps (Fig. 4b). The precision-recall curve showing the trade-off between precision and recall at various cut-off also illustrate that the top-performing teams for most range (recall $\ge 0.5$–1.0, Fig. 4c) are HoLLYS_ETRI and JIN_ZJU teams. However, this is not true for below range values for JIN_ZJU for all datasets, while AIM_CityU team showed consistent performance for all ranges (Fig. 4c and Supplementary Fig. 3). A comparable observation can be identified about the deviation scores in the assessment of generalisability (Fig. 4d-e). Even though the proposed single-stage YOLO-based detector by JIN_ZJU provided a second best score on the seen data sequence (data 3), it showed the highest performance drop (Fig. 4f) to the unseen sequence data (data 4) of nearly above 25% on AP (Supplementary Table 3).

For the single frame datasets (i.e. both NBI image samples, data 1 and unseen centre WLE image samples, data 2), the methods presented by teams HoLLYS_ETRI and JIN_ZJU outperformed in terms of AP values. The results by both teams on data 1 had an increased difference for AP_mean_ (> 9%), AP_50_ (> 9%) and AP_75_ (> 10%) when compared to the other teams (Supplementary Table 3). Additionally, team HoLLYS_ETRI provided the best AP performance across the different scales. Similarly, on data2, teams HoLLYS_ETRI and JIN_ZJU delivered a high AP value compared to the other teams. However, team AIM_CityU produced comparable results leading them to third place with a small difference of 0.0051 for AP_mean_ score when compared to team HoLLYS_ETRI.

For the seen sequence dataset (Data 3, Supplementary Table 3), team JIN_ZJU preserved the high performance when evaluating the AP_mean_ (i.e. higher than second-best team AIM_CityU by 4.19%) and the AP_75_ (i.e. higher than second-best team HoLLYS_ETRI by 3.29%). Team HoLLYS_ETRI provides the best result for AP_50_ with a difference of 2.10% when compared to AIM_CityU that comes in second place. Furthermore, the method by HoLLYS_ETRI surpassed the results of other teams and baseline methods on the unseen sequence (Data 4) where the second teams take place with a difference of greater than 0.037, 0.04 and 0.055 on AP_mean_, AP_50_ and AP_75_, respectively. In general, results by teams HoLLYS_ETRI, JIN_ZJU and AIM_CityU achieved the best performance even when compared to the baselines method. It can be derived that the three teams HoLLYS_ETRI, JIN_ZJU and AIM_CityU provided a high performance showing a larger area under the curve for all datasets (Supplementary Figure 3), while the baseline method EfficientDetD2 gave the lowest performance, followed by the YOLOv4.

Table 2 shows the ranking of the detection task of the polyp generalisation challenge after calculating the average detection precision, average deviation scores and time. Team AIM_CityU ranks the first place with inference time of 100 ms per frame and lowest deviation scores of dev_g_2-3_ (0.134), dev_g_4-3_ (0.056) and dev_g (0.093). Followed by team HoLLYS_ETRI in second place with an increased inference time of 690 ms per frame and difference of dev_g_2-3_ (0.078), dev_g_4-3_ (0.426) and dev_g (0.051). But, it secured the top score for average detection with a value of 0.491. Finally, in the third place, team JIN_ZJU takes place with 1900 ms per frame for the inference time and the second-best average detection result of 0.478.

**Table 2:** Ranking of detection task of polyp generalisation challenge.

Team/method	Avg_det $\uparrow$	Avg. deviation scores $\downarrow$				Time $\downarrow$	Rank $\downarrow$
		dev_g_1-3_	dev_g_2-3_	dev_g_4-3_	dev_g	(in ms)	
AIM_CityU^18^	0.450	0.089	**0.134**	**0.056**	**0.093**	**100**	**1**
GECE_VISION^20^	0.384	**0.056**	0.253	0.069	0.126	320	5
HoLLYS_ETRI^24^	**0.491**	0.122	0.212	0.098	0.144	690	**2**
JIN_ZJU^19^	**0.478**	0.062	0.230	0.091	0.128	1900	3
YOLOv4^27^	0.316	0.099	0.178	0.060	0.112	13	6
RetinaNet (ResNet50)^28^	0.320	**0.031**	**0.086**	**0.040**	**0.052**	27	4
EfficientDetD2^29^	0.298	0.058	0.173	0.078	0.103	200	7

Average precision across all test splits is provided as Avg_det. Deviation scores are calculated between the test data 3 w.r.t. data 1 (dev_g_1-3_), data 2 (dev_g_2-3_) and data 4 (dev_g_4-3_). An average deviation score dev_g is computed by averaging the computed deviations for each data. Test execution time is provided in ms. Finally, a rank column is used to provide an average rank based on the computed ranks for each Avg_det, dev_g and time. Top-two values for each metric are highlighted in bold.

$\uparrow$: best increasing         $\downarrow$: best decreasing.

### Aggregated performance and ranking on segmentation task

**Figure 5:**
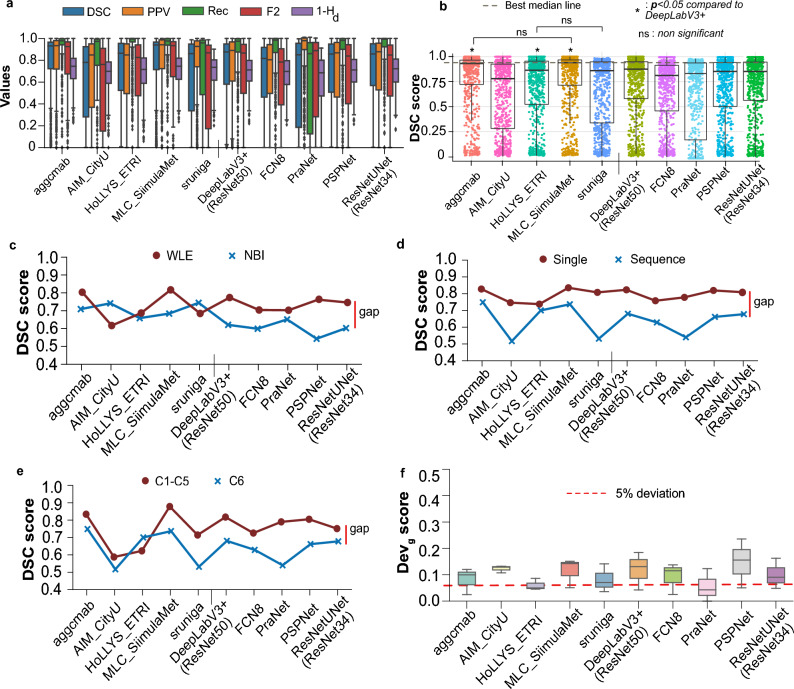
Generalisation assessment on segmentation task: (**a**) box plots for all segmentation metrics (dice coefficient, DSC; precision, PPV; recall, Rec; F2, type-II error; and Hausdorff distance, Hd) used in the challenge for all test data samples. (**b**) Boxplots representing descriptive statistics overall cases (median, quartiles and outliers) are combined with horizontally jittered dots representing individual data points in all test data. A red line represents the best median line. It can be observed that teams aggcmab and MLC_SimulaMet have similar results and with Friedman–Nemenyi post-hoc *p* value $< 0.05$, denoting a significant difference with the best-performing baseline DeepLabv3+ method. (**c**) White light endoscopy, WLE versus narrow-band imaging, NBI, (**d**) single versus sequence data, (**e**) seen centres, C1–C5 versus unseen centre, C6 and (**f**) deviation scores. The red line in (**c**–**e**) represents performance gaps among all the methods. It can be observed that the gaps are variable for different data for most methods. However, for some teams, the performance gaps are smaller than the baseline while maintaining a higher DSC score. Finally, for (**e**), a dashed line with 5% deviation is drawn, showing three out of 5 team methods aligning with this line while only PraNet and ResNetUNet aligned with this demonstrating lower deviation in DSC scores when compared with the aggregated deviation of test sets w.r.t unseen test data 3.

Figure 5a demonstrate the boxplots for each teams and baseline methods. It can be observed that the median values for all area-based metrics (dice, precision, recall and F2) are above 0.8 for most teams when compared on all 777 test samples. However, a greater variability can be observed for all teams and baselines represented by a large number of outlier samples. Only marginal change can be seen for the mean distance-based normalised metric ($1-H_d$) for which top teams have higher values as expected. On observing closely only the dice similarity metric in Fig. 5b where dot and box plots are provided, teams MLC_SimulaMet and aggcmab obtained the best scores demonstrating least deviation and with most samples concentrated in the interquartile range. It can be observed that paired aggcmab and MLC_SimulaMet; DeepLabV3+(ResNet50) and ResNetUNet(ResNet34); and HoLLYS_ETRI and PSPNet have similar performances since their quartiles Q1, Q2, and Q3 scores are very close to each other. Although the mean DSC score of team aggcmab is slightly higher than the MLC_SimulaMet, there was no observed a statistically significant difference between these two teams. However, both of these teams reported significant differences with $p<0.05$ when compared to the best performing baseline DeepLabV3+(ResNet50).

For data 1 (NBI single frame images, Supplementary Table 4), the method suggested by teams sruniga and AIM_CityU outperformed against the other team’s baseline methods in terms of JC (> 0.65), DSC (> 0.74) and F2 (> 0.73). The team sruniga had an outstanding performance in segmenting fewer false-positive regions achieving a PPV result of 81.52 %, which is higher than other methods by at least 5%. Nevertheless, the top recall value for team MLC_SimulaMet and HoLLYS_ETRI (> 0.86) proves their ability in detecting more true positive regions. The accuracy results on this data were comparable between all teams and baseline methods ranging from 95.78 to 97.11% with the best performance by team AIM_CityU. For data 2 (i.e., white light, unseen centre, single frames), the methods developed by teams MLC_SimulaMet and aggcmab produced the top values for JC (> 0.77), DSC (> 0.82) and F2 (> 0.81) with comparable results between two teams (second row of the data type column, Supplementary Table 4). The PPV value was maintained with the method proposed by team sruniga (i.e. as discussed for data 1) with value of 0.8698 ± 0.21 followed by team MLC_SimulaMet in second place with a value of 0.8635 ± 0.26. Additionally, the method by team MLC_SimulaMet surpassed the results for all evaluation measures when compared to the other teams and baseline methods on data 3 (third row of the data type column, Supplementary Table 4). Moreover, the method proposed by team aggcmab comes in second place with more the 5% reduction of results for the JC, DSC and HDF. For this dataset, the baseline method DeepLabV3+ (ResNet50) showed improved performance compared to results on previously discussed data (i.e. data 1 and data 2), where it acquires second place for the F2 and accuracy with a result of 82.66% and 95.99% respectively. On Data 4 (unseen centre sequence, last row of the data type column in Supplementary Table 4) methods by teams MLC_SimulaMet and aggcmab produce the best results for most of the evaluation measures JC (> 0.68), DSC (> 0.73), F2 (> 0.71), ACC (> 0.97) and HDF (< 0.34). Generally, throughout the evaluation process for all tables on the different datasets, team sruniga provided a high PPV value on data 1, data 2 and data 4. Furthermore, the baseline methods showed low performance in different segmentation metrics compared to the methods proposed by the participants, especially with data 1, data 2 and data 4. Supplementary Figure 4 represents the proportion of each test data split contributing to the ranking of each team and baseline methods. It can be observed that team MLC_SimulaMet and team aggcmab were consistently ranked higher (% of data samples aligning around top rank 1) across almost all test data splits except for data 1, in which case most samples for team sruniga and team AIM_CityU were ranked better in comparison. Similarly, Supplementary Figure 6a demonstrates that around 28.33% of data 1, 21.25% of data 2, 21.66% of data 3 and 31.5% of data 4 has DSC equal or lower than 0.50. Polyp size variabilitiy for each image sample showed that the highest metric values were those with more obvious protruded polyps (Supplementary Figure 5), while subtle polyps appearing next to the folds were among worse performing samples (refer Supplementary Figure 6b) including image samples with partial polyp views in sequence data were among the flagged worse samples (data 3 and data 4 in Supplementary Figure 6b).

To understand the behaviour of each method for provided test data splits, we plotted DSC values each separately and compared the ability of methods to generalise on these. From Fig. 5c,d it can be observed that difference in data setting affected almost all methods. It can be observed that there is nearly up to 20% gap in performance of the same methods when tested on WLE and NBI. In the same way, this applies to both the single frame and sequence frame scenarios, as well as to the use of previously unseen center data. However, it could be observed that those methods that had very close values (e.g., HoLLYS_ETRI) suffered in achieving higher metric values compared to the other methods. A difference was also observed in single frames from the seen centre (data 3) sequence to the unseen centre (data 4) sequence (Fig. 5e).

To assess generalisability of each method, we also computed deviation scores for semantic segmentation referred to as dev_g (see Table 3; Fig. 5f). For this assessment, team aggcmab ranked first on both average segmentation scores R_seg_ and deviation score R_dev_. Even though team sruniga was only third on R_seg_, they were second on R_dev_ and ranked at the 1st position for their computation time with an average inference time of only 17 ms per second. Team MLC_SimulaMet only was ranked third due to their large computational time of 120 ms per frame and more significant deviations (lower generalisation ability). Additionally, Supplementary Figure 7 illustrates that imperfections in colonoscopic frames pose a challenge to the efficacy of various approaches since most of them are susceptible to producing a higher number of false positives. Our illustration shows that team aggcmab and team MLC_SimulaMet that used ensemble methods provided the least false positives. We provide the results of teams with performance below the baseline and poor ranking compared to the top five teams analysed in the paper in the Supplementary Table 5 for completeness. It is to be noted that these teams were selected in round 3 of the challenge as well but have not been analysed in this paper due to their below baseline scores.

**Table 3:** Ranking of segmentation task of polyp generalisation challenge: ranks are provided based on (a) semantic score aggregation, R_seg_; (b) average deviation score, R_dev_; and (c) overall ranking (R_all_) that takes into account R_seg_, R_dev_ and time.

Team/method	Average Seg_score $\uparrow$			Average Dev_score $\downarrow$			Time $\downarrow$ (ms)	R_seg_ $\downarrow$ (avg.)	R_dev_ $\downarrow$ (avg.)	R_all_ $\downarrow$ (avg.)
	Data 1	Data 2	Data 4	dev_g_1-3_	dev_g_2-3_	dev_g_4-3_				
Aggcmab^21^	0.746	**0.849**	**0.788**	0.119	**0.024**	0.099	107	**1**	**1**	**1**
AIM_CityU^18^	**0.762**	0.777	0.589	0.128	0.132	0.107	80	4	3	5
HoLLYS_ETRI^24^	0.714	0.777	0.746	**0.045**	0.086	**0.049**	84	5	**1**	4
MLC_SimulaMet^22^	0.741	**0.858**	**0.781**	0.151	0.051	0.142	120	**2**	3	3
Sruniga^23^	**0.771**	0.830	0.611	**0.035**	0.070	0.141	**17**	3	2	**2**
Baselines										
DeepLabV3+ (ResNet50)^30^	0.669	0.838	0.726	0.184	0.042	0.131	19	NA	NA	NA
PSPNet^30,31^	0.593	0.832	0.710	0.235	0.050	0.155	45	NA	NA	NA
FCN8^32^	0.651	0.787	0.684	0.137	**0.024**	0.115	27	NA	NA	NA
ResNetUNet-ResNet34^33^	0.658	0.823	0.729	0.162	0.048	**0.090**	**13**	NA	NA	NA

For ties in the final ranking (R_all_), segmentation score is taken into account. For time, ranks are provided into three categories: teams with $< 50$ ms, between 50–100 ms and $> 100$ ms. Top-two values for each metric are highlighted in bold.

## Discussion

While polyp detection and segmentation using computer vision methods, in particular deep learning, have been widely studied in the past, rigorous assessment and benchmarking on the centre-wise split, modality split and sequence data have not been comprehensively studied. Our EndoCV2021 edition challenged participants to address the generalisability mentioned above issues in polyp detection and segmentation methods on a multicentre dataset.

For polyp detection and localisation, 3/4 teams chose feature pyramid-based network architectures that use regions to localise objects of interest within an image. In contrast, one team (JIN_ZJU) used the YOLOV5 ensemble paradigm based on multiple differently sized grid boxes but is a faster model than the former. Unlike most other team methods that require anchors to detect various objects of different scales and overlap, team AIM_CityU used an anchor free fully convolution, one-stage object detection (FCOS) method. HoLLYS_ETRI mainly focused on accuracy and used an ensemble to train five different models, i.e., one model per centre, and an aggregated model output was devised for the test inference. Even though the HoLLYS_ETRI team showed top ranking on the average detection score on almost test data splits (Supplementary Table 3, and average precision scores in Fig. 4a–c), the observed detection speed of 690 ms and the high deviation in generalisation scores only put them on the second rank (see Table 2). On the contrary, AIM_CityU team, with their anchor free single stage network, performed consistently well in almost all data with the fastest inference of 100 ms and the slightest deviation scores (see Fig. 4d,e; Table 2) between teams. Anchor free methods perform better than other methods on sequence data (2nd best for both seen and unseen sequences) because FCOS-based detection methods are less sensitive to the dynamic scene changes as they do not depend on pre-defined anchor boxes and region proposals. Thus, it can be concluded that anchor free detection methods can better generalise compared to methods that require anchors in heterogeneous multicentre datasets. This is strictly true as the polyp sizes (in pixels) in the dataset is varied (Fig. 1b) and also the image sizes ranged from $388 \times 288$ pixels to $1920 \times 1080$ pixels. Also, for the video sequences anchor, free methods are more suitable as polyps occurrences are observed at multiple visual scales.

Since all methods trained their algorithm on single-frame images, detection scores for all methods are relatively higher for the data 2 (WLE-single, Supplementary Table 3), compared to the other data categories, although they came from unseen data centre 6. However, performance drop can be observed for both seen (centres, C1–C5, data 3) and unseen (centre, C6, data 4) sequence data that consisted of WLE images only. In addition, change in modality has a detrimental effect on the performance for all methods, even on single frames (see for data 1, NBI-single, Supplementary Table 3). A similar drop in performance (nearly 25% difference in average precision compared to the seen sequence) was observed for the unseen centre sequence test data (data 4). As a result, the average deviation scores in detection for computed for each team were above 10% deviation line for the overall aggregated overall deviation scores (Fig. 4e) and significantly lower scores compared to seen sequence (data 3) and unseen sequence (data 4) test splits (Fig. 4f). Thus, the methods trained on single frames produce sub-optimal and inconsistent detection in videos as image-based object detection cannot leverage the rich temporal information inherent in video data. The scenario worsens when applied to a different centre to that on which it was trained. To address the limitation of generalization to sequence data, it is possible to employ Long Short-Term Memory (LSTM) based techniques, which effectively preserve temporal information to encourage the improvement of predictions^34^.

For segmentation task, while most teams used ensemble technique targeting to win on the leaderboard (MLC_SimulaMet, HoLLYS_ETRI, aggcmab), there were some teams who worked towards model efficiency network (e.g., team sruniga) or modifications for faster inference and improved accuracy (e.g., team AIM_CityU). Lightweight model using HarDNet68 backbone with aggregated maps across scales (team sruniga) and use of multi-scale feature fusion network (HRNet) with low-rank disentanglement by team AIM_CityU outperformed all other methods on narrow-band imaging modality (data 1), including the baseline segmentation methods (Supplementary Table 4). These methods showed acceptable performance for single frames on unseen data (data 2, WLE-single) as well. However, on sequence data (both for seen sequence data 3 and unseen sequence data 4), both of these methods performed poorly compared to ensemble-based techniques (see Fig. 5). Several networks conjoint by MLC_SimulaMet and dual UNet network used by the team aggcmab have the disadvantage of large inference time (nearly six times higher than the fastest method). However, it can also be observed that these teams provided more robust output in the sequence data (Supplementary Figure 7) where most other methods were affected by frame corruption giving more false positives. It is essential to note that this is major bottleneck of most deep learning methods for polyp detection and segmentation. Similarly, when it came to capturing the size-variability of polyps ensemble segmentation models are more appropriate than using a single model (see Supplementary Figure 5. So the takeaway message is that on single-frame data, multi-scale feature fusion networks perform better irrespective of their modality changes. This is without requiring the ensemble of the same or multiple models for inference which ideally increases both model complexity and inference time. However, on sequence data and capturing varied-sized polyps, we advise incorporating temporal information propagation in the designed networks. Methods with ensemble models are more desirable to eliminate false positives in these scenarios, but with more computational time. Furthermore, to improve model generalisation on unseen modality, domain adaptation techniques can be applied^12^.

HoLLYS_ETRI used instance segmentation approach with five separate models trained on C1–C5 training data separately. It can be observed that this scheme provided better generalisation ability in most cases leading to the least deviation on average dice score (see Fig. 5f). Also, it is only team that obtains better results in C6 compared to C1–C5 and very comparable result between single and sequence frames. However, reported dice metric values were lower than most methods, especially ensemble (MLC_SimulaMet) and cascaded (aggcmab) techniques targeted towards higher accuracy but are less generalisable (consistency in test inference across multiple data categories). This is also evident in Supplementary Fig. 4, where proportion of samples from data 1 for top-performing teams aggcmab and MLC_SimulaMet are only ranked on the third and fourth test splits. Therefore, it can be concluded that pretext tasks can lead to improved generalisability. However, to boost model accuracy, modifications are desired that could include feature fusion blocks and other aggregation techniques.

All the top methods developed in our crowd-sourcing event surpassed widely used baseline deep learning methods for detection task (Table 2) and segmentation task (Table 3) by large margins. For segmentation task, 3 out-of 5 devised methods showed significant difference ($p<0.05$) with the best performing current baseline method (Fig. 5b). However, not all guaranteed robustness and some of these “accurate” methods provided less than ten frames-per-second, which is 3–6 times less than the colonoscopy acquired videos. Final takeaway message from our experimental findings is that devising a model specific to the polyps require an understanding of the data, robustness tests and real-time inference capability for clinical usability. The reliance and confidence in the technology can only be guaranteed upon thoroughly testing the developed method under actual clinical procedure scenarios. While method accuracy is important, the consistency of the method to perform equally well in different settings that are clinically required is important.

Clinical adoption of the methods requires generalizability assessment on different clinical modalities and multi-population datasets as conducted in this study. Our study demonstrated that no single deep-learning method could improve the accuracy and robustness of baseline models alongside real-time performance. Most methods use an ensemble of the models that provide higher accuracy but a sacrifice in speed. Model performance gaps are eminent in current deep learning techniques. To strengthen these, more diverse datasets are required to be devised and trained on. Similarly, visual cues such as polyp shapes could be used to improve the robustness of methods^11^. Video polyp segmentation techniques using transformers can be used to improve the model inferences^35^.

## Conclusion

In this work, we presented an extensive dissection of deep learning methods for polyp detection and segmentation devised by several top participants in the crowd-sourcing initiative. A comprehensive approach is forwarded to assess the usability of deep learning models in routine clinical colonoscopy. Our experimental design provided holistic comparisons on a diverse six centre dataset. While most methods provided an improvement over widely-used current baseline methods, the method design of teams adversely impacted algorithmic robustness and real-time capability, mainly when provided unseen sequence data and different modalities. A better trade-off in inference time and generalisability can be the key takeaways for further development in this area. Thus, we demonstrated the need for generalisable methods to tackle real-world clinical challenges. Experimental-based hypotheses were derived after studying the strategies for developing the suggested methodology of the highly-ranked teams, key findings can be concluded as follows: (a) methods that use anchor-free algorithms generalise more effectively; (b) methods proposed for sequence data can benefit from the incorporation of temporal information to improve the prediction; (c) model performance can be improved by techniques like multi-scale feature fusion, fusion blocks, and other aggregation algorithms and understanding the data; and (d) having the proficiency to construct real-time inferences for clinical usability is necessary for creating a model. Accordingly, future research towards innovating more practical strategies that can work effectively in multi-centre data and diverse modalities often used in clinical colonoscopy procedures. In addition, incorporating temporal relationships in the network designs can be vital for improvement of both accuracy and robustness of polyp detection and segmentation methods in colonoscopy. In future work, we aim to develop a larger dataset with video sequences directly both for training and test which will be valuable for assessing deep learning methods for clinical usability.

### Supplementary Information

Supplementary Information.

## Data Availability

To access the complete dataset, users are requested to create a Synapse account (https://www.synapse.org/) and then the compiled dataset can be downloaded at (https://www.synapse.org/#!Synapse:syn45200214). For more details on the data please refer to^16^.

## Code availability

To help users with the evaluate the generalizability of detection and segmentation method a code is available at: https://github.com/sharib-vision/EndoCV2021-polyp_det_seg_gen. The code also consists of inference codes that to assist in centre-based split analysis. Benchmark codes of the polypGen dataset with provided training and validation split in this paper for segmentation is also available at: https://github.com/sharib-vision/PolypGen-Benchmark.git. All the method codes for detection (d1-d4) and segmentation (s1–s8) are also available at different GitHub repositories provided (see column Code of Table 1).
