# Supplementary material for: Assessing generalisability of deep learning-based polyp detection and segmentation methods through a computer vision challenge
Source: Sci Rep. 2024 Jan 23;14:2032. doi: 10.1038/s41598-024-52063-x (PMC10805888; doi:10.1038/s41598-024-52063-x)
Supplement: Supplementary file 1 — Supplementary Information. [file 41598_2024_52063_MOESM1_ESM.pdf]

Supplementary material: Assessing generalisability of deep learning-based polyp detection and segmentation methods through a computer vision challenge

Sharib Ali[1,2,3,*], Noha Ghatwary[5], Debesh Jha[6,7], Ece Isik-Polat[13], Gorkem Polat[13], Chen YANG[14], Wuyang LI[14], Adrian Galdran[15], Miguel-Ángel González Ballester[15], Vajira Thambawita[6], Steven Hicks[6], Sahadev Poudel[16], Sang-Woong Lee[16], Ziyi Jin[17], Tianyuan Gan[17], ChengHui Yu[18], JiangPeng Yan[19], Doyeob Yeo[20], Hyunseok Lee[21], Nikhil Kumar Tomar[22], Mahmood Haithmi[23], Amr Ahmed[23], Michael A. Riegler[6,7], Christian Daul[24], Pål Halvorsen[6,25], Jens Rittscher[2], Osama E. Salem[12], Dominique Lamarque[11], Renato Cannizzaro[10,†], Stefano Realdon[9,†], Thomas de Lange[8,26,27,†], and James E. East[3,4,†]

[1]School of Computing, University of Leeds, LS2 9JT, Leeds, United Kingdom
[2]Institute of Biomedical Engineering, Department of Engineering Science, University of Oxford, OX3 7DQ, Oxford, United Kingdom
[3]Oxford National Institute for Health Research Biomedical Research Centre, OX4 2PG, Oxford, United Kingdom
[4]Translational Gastroenterology Unit, Nuffield Department of Medicine, Experimental Medicine Div., John Radcliffe Hospital, University of Oxford, OX3 9DU, Oxford, United Kingdom
[5]Computer Engineering Department, Arab Academy for Science and Technology, 1029, Alexandria, Egypt
[6]SimulaMet, 0167 Oslo, Norway
[7]Department of Computer Science, UiT The Arctic University of Norway, Hansine Hansens veg 18, 9019 Tromsø, Norway
[8]Medical Department, Sahlgrenska University Hospital-Mölndal, Blå stråket 5, 413 45 Göteborg, Sweden
[9]Veneto Institute of Oncology IOV-IRCCS, Via Gattamelata, 64, 35128 Padua, Italy
[10]CRO Centro Riferimento Oncologico IRCCS Aviano Italy, Via Franco Gallini, 2, 33081 Aviano PN, Italy
[11]Université de Versailles St-Quentin en Yvelines, Hôpital Ambroise Paré, 9 Av. Charles de Gaulle, 92100 Boulogne-Billancourt, France
[12]Faculty of Medicine, University of Alexandria, 21131, Alexandria, Egypt
[13]Graduate School of Informatics, Middle East Technical University, 06800 Ankara, Turkey
[14]City University of Hong Kong,Kowloon, Hong Kong
[15]BCN MedTech, Dept. of Information and Communication Technologies, Universitat Pompeu Fabra, 08018, Barcelona, Spain
[16]Department of IT Convergence Engineering, Gachon University, Seongnam 13120, Republic of Korea
[17]College of Biomedical Engineering and Instrument Science, Zhejiang University, Hangzhou 310027, China
[18]Tsinghua Shenzhen International Graduate School, Tsinghua University, Shenzhen 518055, China
[19]Department of Automation, Tsinghua University, Beijing 100084, China
[20]Smart Sensing & Diagnosis Research Division, Korea Atomic Energy Research Institute, 34057, Republic of Korea
[21]Daegu-Gyeongbuk Medical Innovation Foundation, Medical Device Development Center, 427724, Republic of Korea
[22]NepAL Applied Mathematics and Informatics Institute for Research (NAAMII), Kathmandu, Nepal
[23]Computer Science Department, University of Nottingham, Malaysia Campus, 43500 Semenyih, Malaysia
[24]CRAN UMR 7039, Université de Lorraine and CNRS, F-54500, Vandœuvre-Lès-Nancy, France
[25]Oslo Metropolitan University, Pilestredet 46, 0167 Oslo, Norway
[26]Department of Molecular and Clinical Medicine, Sahlgrenska Academy, University of Gothenburg, 41345 Göteborg, Sweden
[27]Augere Medical, Nedre Vaskegang 6, 0186 Oslo, Norway
[*]corresponding author: Sharib Ali (s.s.ali@leeds.ac.uk)
[†]these authors contributed equally to this work

Supplementary Figures and Tables

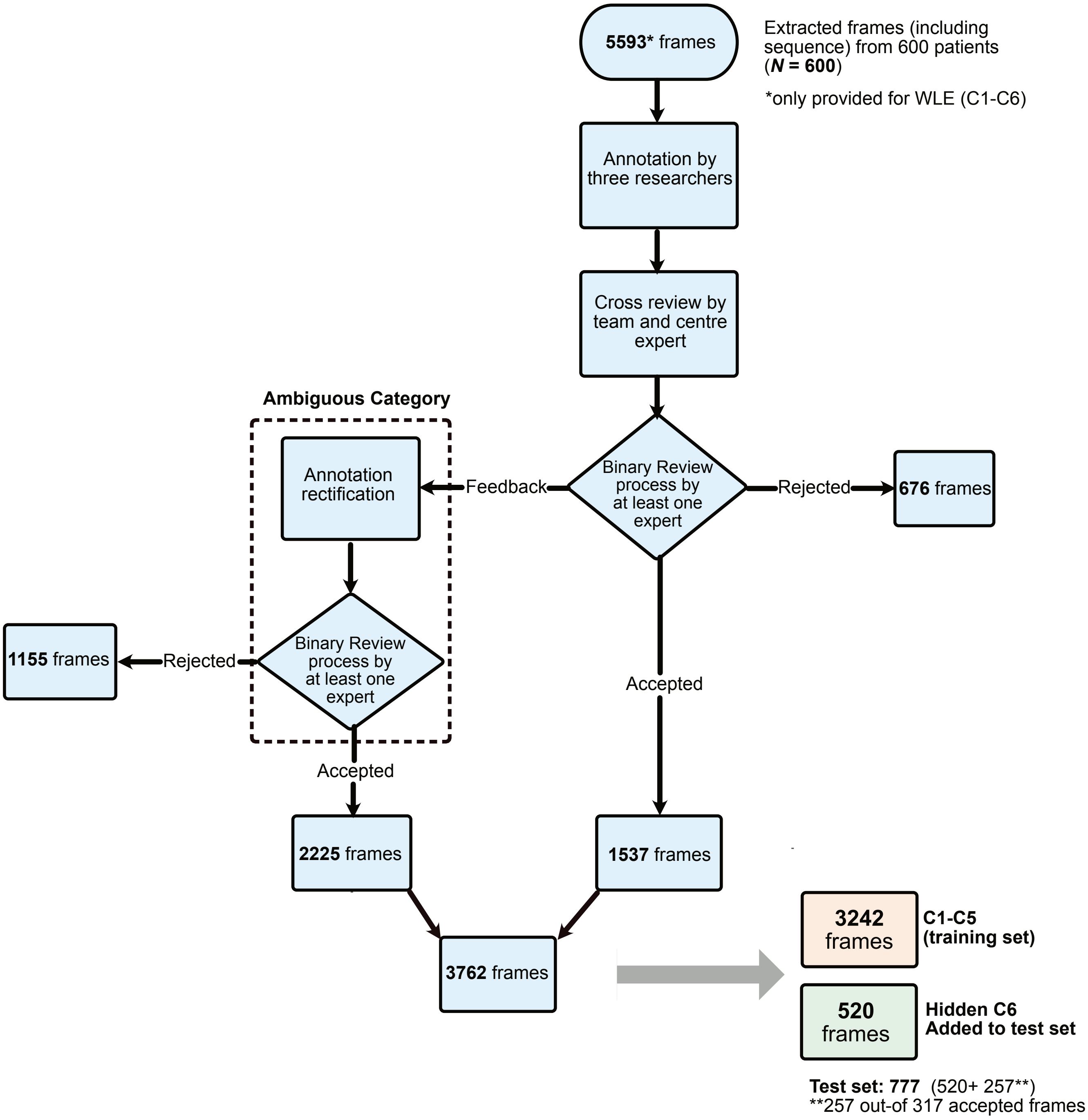

Supplementary Figure 1. Annotation workflow: 600 patients ($N = 600$) data was used that consisted of both videos and frames. First 5593 relevant frames for polyp detection and segmentation were extracted. These frames comprised of both single and sequence data. For details please see Fig. 1 (main manuscript). Review of annotations was done by at least one expert and the frames were either re-labeled or immediately rejected. A second review was conducted by at least one expert. Here expert refers to a senior consultant gastroenterologist. Overall, 3762/5593 frames were annotated of which 520 frames from center 6 was directly embedded in the test set. For testing set, a similar strategy was taken for which 257 samples out-of 317 samples were accepted during the review phase.

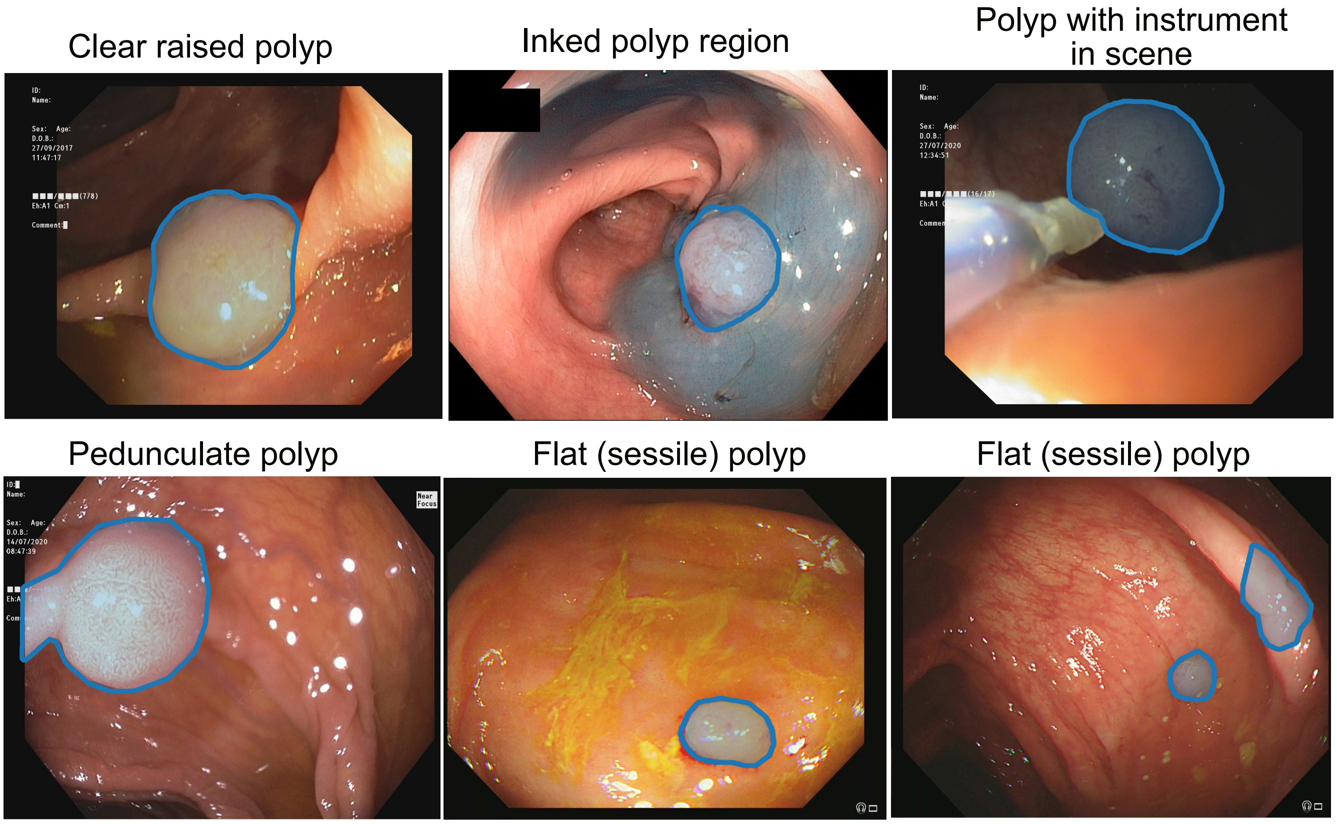

Supplementary Figure 2. Sample annotated images following the annotation protocol. Clear raised polyp, polyp with the inked region, polyp with the instrument in the scene, pedunculate polyp and flat (sessile) polyps are illustrated.

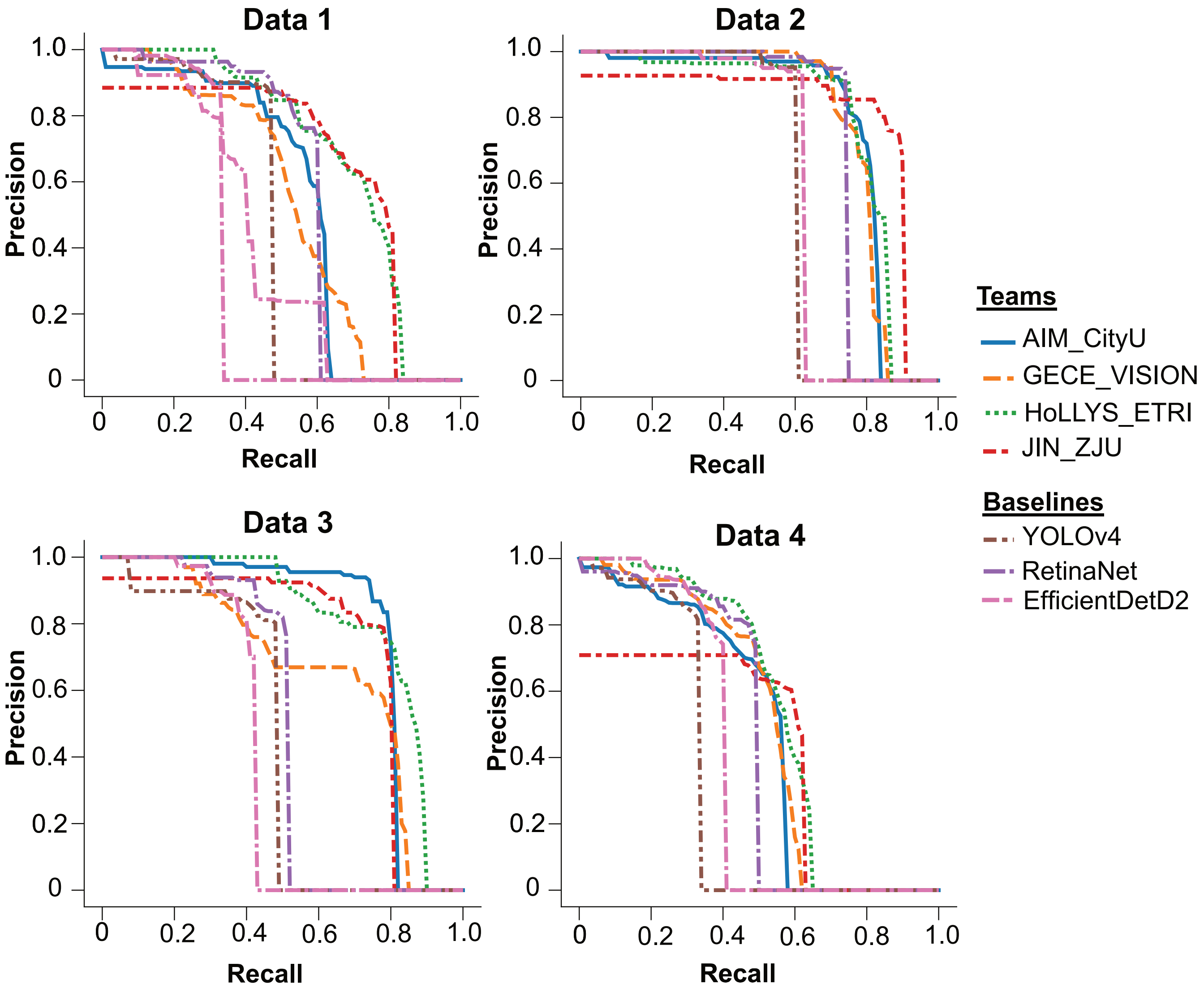

Supplementary Figure 3. Precision-recall (PR) curve for detection task. Precision and recall of the participants and baseline methods are evaluated at intersection-over-union threshold of 0.5. Data 1 consisting of unseen modality with NBI data, data 2 comprising of single frames of unseen center C6, data 3 consisting of mixed seen center (C1-C5) sequence data and data 4 included sequence data from unseen center C6 are used. Interpolated precision at at a certain recall level is plotted. Higher area-under the PR curve denotes better performance.

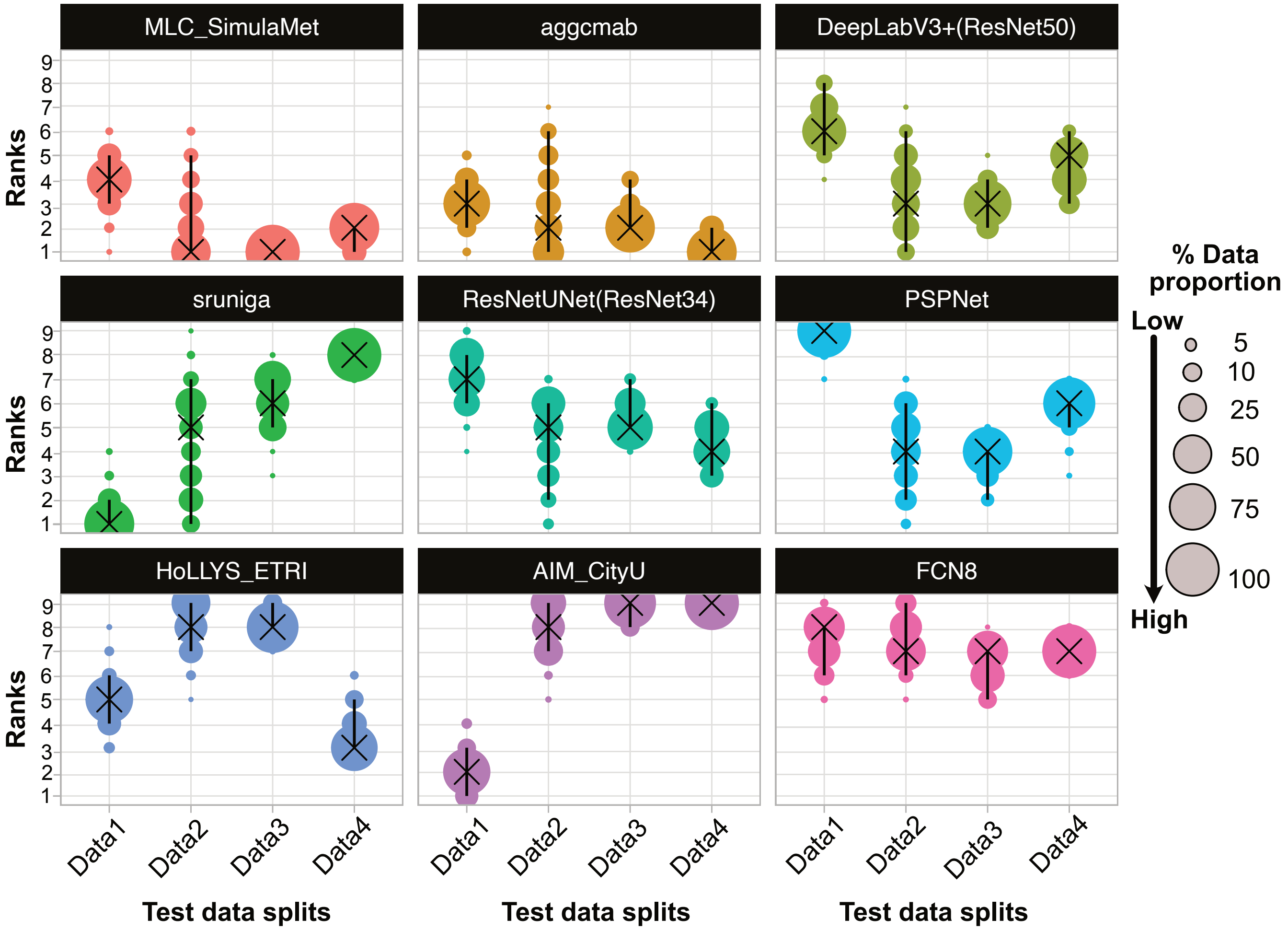

Supplementary Figure 4. Algorithmic rank-based on across bootstrap test data splits are displayed for each team and baseline methods. Different circle sizes signifies how much proportion (in %) of each data contribute to the different rankings (larger the blob size (1%-100%), greater the percentage with 100% be the largest circle). Here, for ranking we have only considered dice similarity coefficient values.

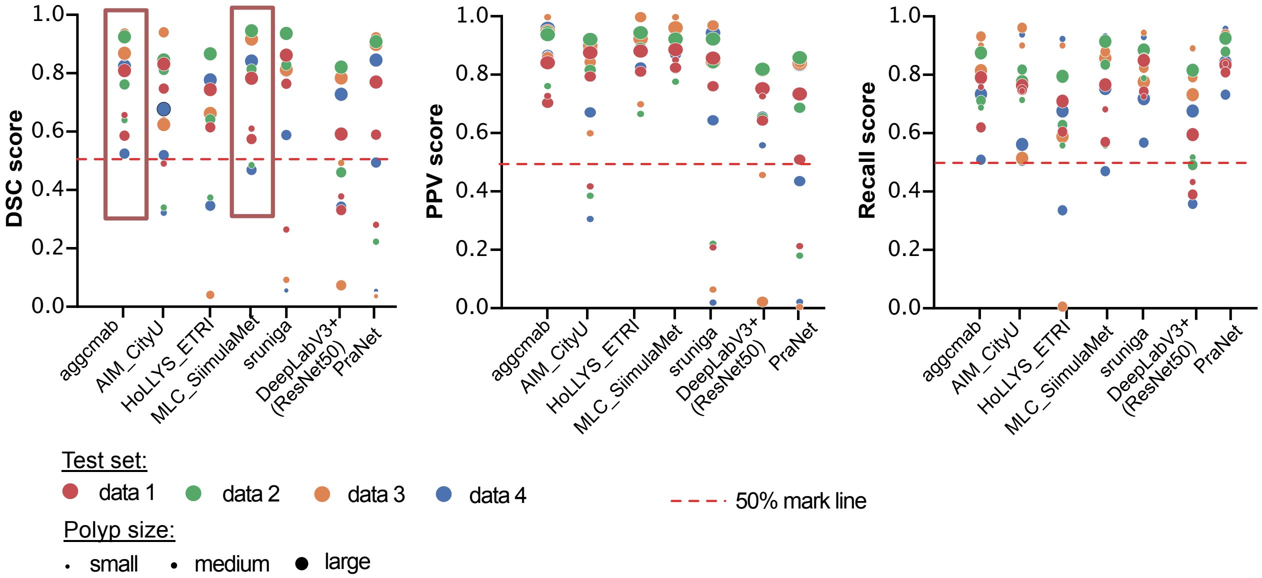

Supplementary Figure 5. Size-based semantic segmentation analysis. Dice similarity coefficient (DSC), positive predictive value (PPV) and recall values are plotted for each team methods and two baseline methods. Red rectangles demonstrates the robust performance for different polyp sizes by two participating teams. The considered polyp sizes are the same that is used for detection (i.e., $\leq 100 \times 100$ for small, and $> 200 \times 200$ large and anything in between is medium).

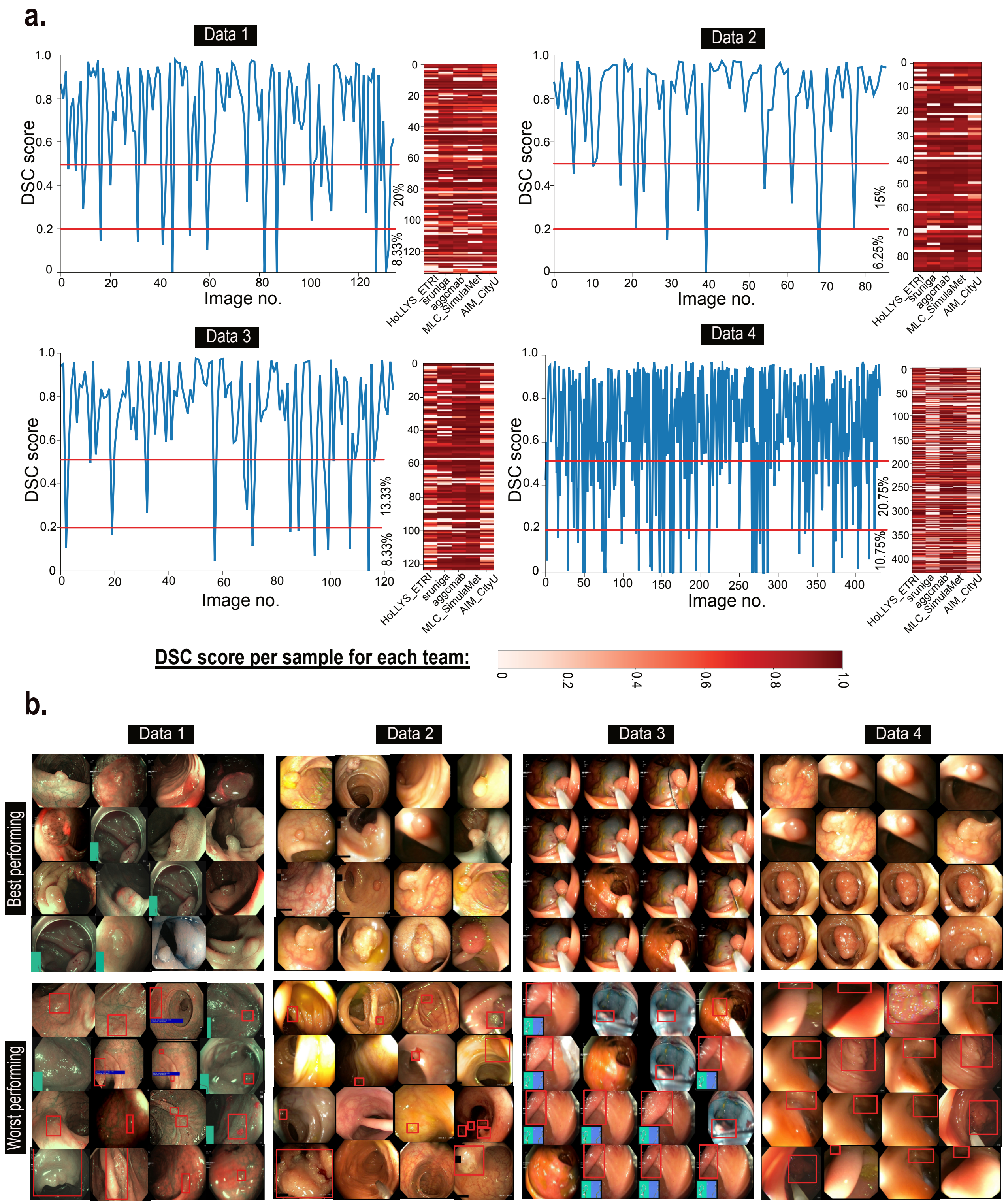

Supplementary Figure 6. Overall team performances on each data based on Dice similarity coefficient, and illustration of the worst and the best-performing samples. a) Dice similarity coefficient for each image sample aggregated for all teams is provided for the test dataset. Additionally, the Histogram bars on the side of each line plot show how much proportion (in %) of each data contributes to the ranking of each team and baseline method. b) Sample frames for worst and best-performing frames in each test data sample. Red areas in the worst performing sample indicate the area with a polyp.

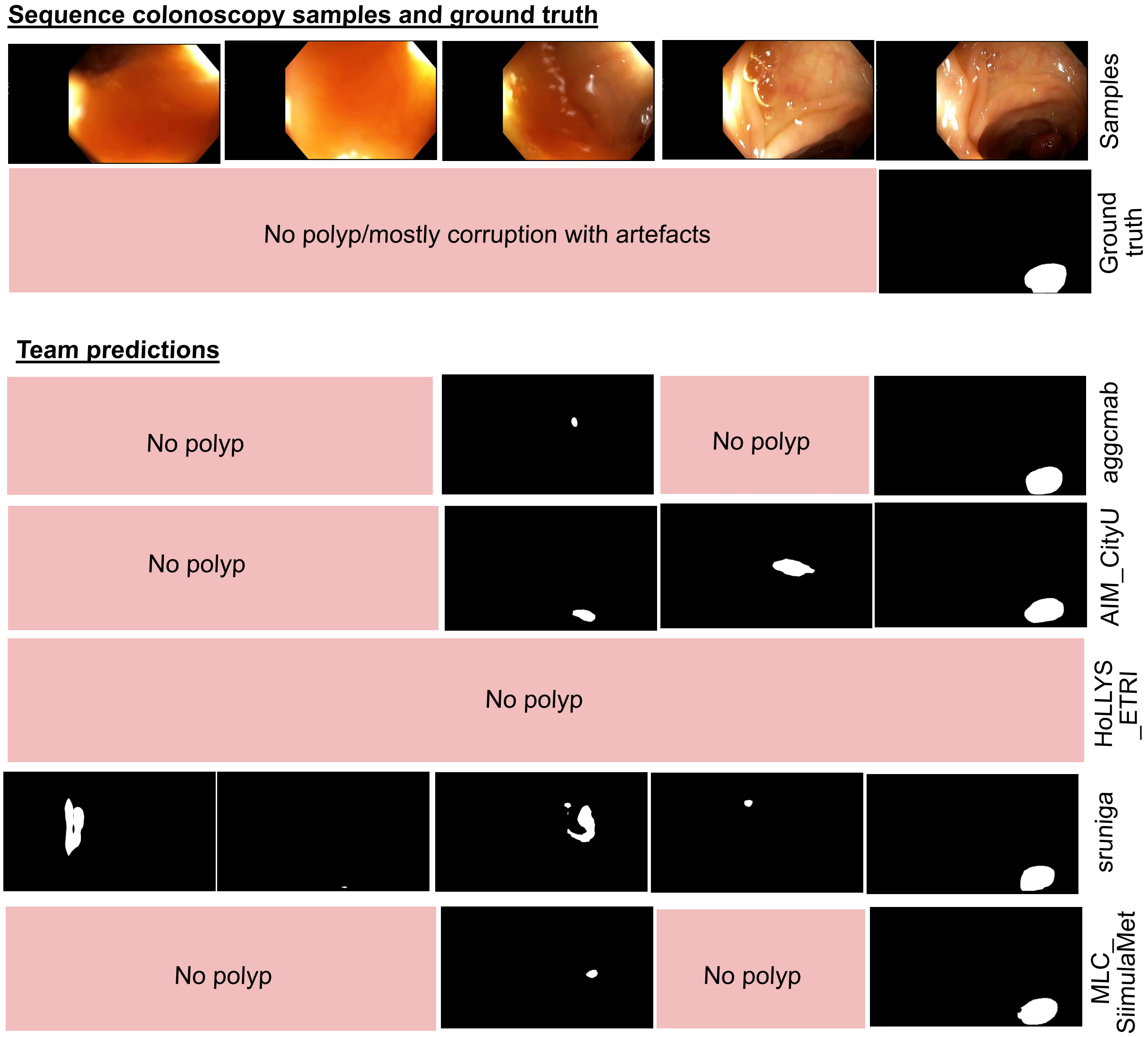

Supplementary Figure 7. Sample images from colonoscopy sequence. Team methods showing variable performance and false detection of samples with artefacts as polyp instances.

Dataset	Findings	# of samples	Resolution	Modality	Study type	Challenge	Availability
CVC-ColonDB[1]	Polyps	380 images† †	574 × 500	WLE	Single	APC	by request•
CVC-ClinicDB[2]	Polyps	612 images†	384×288	WLE	Single	EndoVis	open academic
CVC-VideoClinicDB[3]	Polyps	11,954 images†	384×288	WLE	Single	EndoVis	by request•
EDD2020[4,5]	GI findings with polyps	386 images	variable	WLE, NBI	Multi	EndoCV (2020)	open academic
ETIS-Larib Polyp DB[6]	Polyps	196 images†	1224×966	WLE	Single	EndoVis	open academic
ASU-Mayo polyp database[7]	Polyps	18,781 images†	688 × 550	WLE	Single	EndoVis	by request•
HyperKvasir[8]	GI findings with polyps	110,079 images & 374 videos	720 × 576 to 1920 × 1072	WLE	Single	NA	open academic
Kvasir-SEG[9]	Polyps	1000 images†	332×487 - 1920×1072	WLE	Single	Medico MedAI	open academic
GastroVision[10]	GI findings	8000 frames	720 × 576 to 1920 × 1072	WLE, NBI	Multi	NA	open academic
SUN Colonoscopy Video Database[11]	Polyp non-polyp	158,690 frames	1158 × 1008 to 1240 × 1080	WLE	Single	NA	by request
Endomapper[12]	Endoscopies	96 videos	No info.	WLE	Single	NA	by request
PICCOLO[13]	Polyps	3,433 frames	No. info	WLE NBI	Single	NA	by request
Polypset (KUMC)[14]	Polyps	37,899 frames	592 × 464 to 768 × 576	WLE	Multi	NA	open academic
PolypGen[15]	Polyp non-polyp	3446 images including sequence data†	384 × 288 to 1920 × 1080	WLE, NBI (test)	Multi	EndoCV (2021)	open academic

†Including ground truth segmentation masks ‡Contour ⋄Video capsule endoscopy •Not available anymore or unknown
♣Medical atlas for education with several low-quality samples of various GI findings
APC: Automatic polyp classification; EndoVis: MICCAI Endoscopic vision challenge; EndoCV: IEEE ISBI Endoscopic challenge

Supplementary Table 1. An overview of existing gastrointestinal lesion datasets including polyps

Supplementary Table 2. Data collection information for each center: Data acquisition system and patient consenting information.

Centers	System info.	Ethical approval	Patient consent type	Recording system
Ambroise Paré Hospital, Paris, France	Olympus Exera 195	IDRCB: 2019-A01602-55	Endospectral study	NA
Istituto Oncologico Veneto, Padova, Italy	Olympus endoscope H190	Exempted†	Generic patients consent	ENDOX, TESImaging
Centro Riferimento Oncologico, IRCCS, Italy	Olympus VG-165, CV180, H185	Exempted†	Generic patients consent	Exempted†
Oslo University Hospital, Oslo, Norway	Olympus Evis Exera III, CF 190	Exempted†	Written informed consent	Pix-E5
John Radcliffe Hospital, Oxford, UK	GIF-H260Z, EVIS Lucera CV260, Olympus Medical Systems	REC Ref: 16/YH/0247	Universal consent	MediCapture
University of Alexandria, Alexandria, Egypt	Olympus Exera 160AL, 180AL	Exempted†	Written informed consent	NA

† Approved by the data inspectorate. No further ethical approval was required as it did not interfere with patient treatment

Supplementary Table 3. Team results for the detection task with average precision AP computed at IoU thresholds 50 (AP_{50}), 75 (AP_{75}), and [0.50 : 0.05 : 0.95] mean AP (AP_{mean}). Size wise AP values are also presented. Top-two values for each metric are highlighted in bold.

Data type	Teams/Method	Average precision, AP			AP across scales		
		AP_{mean}	AP_{50}	AP_{75}	AP_{small}	AP_{medium}	AP_{large}
Data 1 (NBI-single)	AIM_CityU[16]	0.351	0.537	0.398	**0.080**	**0.321**	0.408
	GECE_VISION[17]	0.318	0.526	0.349	0.051	0.186	0.398
	HoLLYS_ETRI[18]	**0.474**	**0.693**	**0.552**	**0.130**	**0.396**	**0.550**
	JIN_ZJU[19]	**0.446**	**0.658**	**0.498**	0.038	0.296	**0.586**
	YOLOv4[20]	0.309	0.447	0.372	0.068	0.254	0.371
	RetinaNet (ResNet50)[21]	0.314	0.562	0.267	0.047	0.223	0.381
	EfficientNet-D2[22]	0.201	0.309	0.227	0.029	0.159	0.241
Data 2 (WLE-single)	AIM_CityU[16]	0.573	0.784	0.605	0.279	0.483	**0.659**
	GECE_VISION[17]	0.532	0.785	0.535	0.155	0.459	0.623
	HoLLYS_ETRI[18]	**0.578**	**0.791**	**0.681**	**0.385**	**0.498**	0.655
	JIN_ZJU[19]	**0.604**	**0.809**	**0.664**	**0.307**	**0.614**	**0.721**
	YOLOv4[20]	0.419	0.599	0.463	0.000	0.334	0.523
	RetinaNet[21] (ResNet50)	0.407	0.735	0.461	0.000	0.274	0.524
	EfficientNet-D2[22]	0.420	0.613	0.464	0.000	0.382	0.512
Data 3 (seen seq.)	AIM_CityU[16]	**0.529**	**0.780**	0.548	**0.003**	**0.404**	0.578
	GECE_VISION[17]	0.372	0.658	0.384	0.005	0.026	0.436
	HoLLYS_ETRI[18]	0.528	**0.797**	**0.575**	**0.017**	0.024	**0.599**
	JIN_ZJU[19]	**0.552**	0.725	**0.595**	0.000	**0.151**	**0.651**
	YOLOv4[20]	0.298	0.436	0.354	0.000	0.000	0.328
	RetinaNet (ResNet50)[21]	0.312	0.489	0.356	0.000	0.252	0.341
	EfficientNet-D2[22]	0.293	0.403	0.375	0.000	0.075	0.323
Data 4 (unseen seq.)	AIM_CityU[16]	**0.346**	0.472	**0.376**	0.000	0.272	0.522
	GECE_VISION[17]	0.314	**0.499**	0.330	0.000	0.278	0.472
	HoLLYS_ETRI[18]	**0.384**	**0.540**	**0.432**	**0.000**	**0.309**	**0.580**
	JIN_ZJU[19]	0.309	0.425	0.330	**0.000**	**0.315**	**0.656**
	YOLOv4[20]	0.236	0.311	0.281	0.000	0.208	0.349
	RetinaNet (ResNet50)[21]	0.248	0.449	0.265	0.001	0.176	0.385
	EfficientNet-D2[22]	0.278	0.381	0.336	0.000	0.264	0.407

Supplementary Table 4. Team results for the polyp segmentation methods proposed by the participating teams as well as for the baseline methods. All results are given for sets data 1, 2, 3 and 4. Jaccard Index (JC), Dice Similarity Coefficient (DSC), F2-Score (F2), Positive Predictive Value (PPV), Recall, Accuracy (ACC) and Hausdorff dimension (H_d) are provided. Top-two values for each metric are highlighted in bold. Standard deviations in each metric are shown at two decimal places..

Data type	Teams/Method	JC ↑	DSC ↑	F2 ↑	PPV ↑	Recall ↑	ACC ↑	H_d ↓
Data 1, NBI-single	aggcmab	0.634 ± 0.33	0.709 ± 0.33	0.719± 0.34	0.752 ± 0.34	0.804 ± 0.27	0.967 ± 0.05	0.441 ± 0.20
	AIM_CityU	**0.652 ± 0.28**	**0.741 ± 0.28**	**0.733 ± 0.29**	**0.757 ± 0.28**	0.817 ± 0.26	**0.971 ± 0.05**	0.407 ± 0.16
	HoLLYS_ETRI	0.586 ± 0.35	0.658 ± 0.36	0.656 ± 0.36	0.682 ± 0.37	**0.862 ± 0.24**	0.963 ± 0.06	0.435 ± 0.21
	MLC_SimulaMet	0.616 ±0.35	0.684 ± 0.36	0.691 ± 0.37	0.717 ± 0.37	**0.872 ± 0.21**	**0.967 ± 0.06**	0.437 ± 0.21
	sruniga	**0.667 ± 0.31**	**0.744 ± 0.31**	**0.751 ± 0.31**	**0.815 ± 0.27**	0.776 ± 0.30	0.965 ± 0.08	**0.371 ± 0.16**
	DeepLabV3+ (R50)	0.531 ± 0.33	0.621 ± 0.34	0.601 ± 0.36	0.624 ± 0.36	0.832 ± 0.27	0.964 ± 0.06	**0.397 ± 0.17**
	FCN8	0.505 ± 0.32	0.599 ± 0.34	0.603 ± 0.34	0.644 ± 0.35	0.644 ± 0.35	0.957 ± 0.06	0.433 ± 0.17
	PraNet	0.561 ± 0.33	0.651 ± 0.33	0.695 ± 0.32	0.638 ± 0.30	0.828 ± 0.27	0.928 ± 0.10	0.374 ± 0.19
	PSPNet	0.452 ± 0.33	0.543 ± 0.35	0.518 ± 0.36	0.544 ± 0.36	0.766 ± 0.33	0.956 ± 0.05	0.417 ± 0.16
	ResNetUNet (R34)	0.521 ± 0.35	0.602 ± 0.36	0.584 ± 0.38	0.593 ± 0.38	0.855 ± 0.25	0.963 ± 0.05	0.386 ± 0.17
Data 2, WLE-single	aggcmab	**0.770 ± 0.27**	**0.827 ± 0.27**	**0.819 ± 0.28**	0.828 ± 0.27	**0.923 ± 0.16**	0.983 ± 0.04	0.346 ± 0.18
	AIM_CityU	0.672 ± 0.31	0.746 ± 0.31	0.739 ± 0.31	0.775 ± 0.29	0.849 ± 0.26	0.964 ± 0.11	0.312 ± 0.16
	HoLLYS_ETRI	0.670 ± 0.33	0.737 ± 0.33	0.723 ± 0.33	0.742 ± 0.32	0.908 ± 0.21	0.971 ± 0.08	**0.331 ± 0.17**
	MLC_SimulaMet	**0.777 ± 0.26**	**0.835 ± 0.27**	**0.843 ± 0.27**	**0.863 ± 0.26**	0.893 ± 0.17	**0.985 ± 0.02**	**0.397 ± 0.19**
	sruniga	0.744 ± 0.28	0.807 ± 0.28	0.806 ± 0.28	**0.869 ± 0.21**	0.837 ± 0.28	**0.984 ± 0.02**	0.353 ± 0.16
	DeepLabV3+ (R50)	0.754 ± 0.26	0.823 ± 0.25	0.812 ± 0.26	0.808 ± 0.27	0.911 ± 0.17	0.978 ± 0.06	0.362 ± 0.18
	FCN8	0.676 ± 0.29	0.758 ± 0.28	0.746 ± 0.29	0.745 ± 0.30	0.902 ± 0.16	0.973 ± 0.06	0.425 ± 0.20
	PraNet	0.709 ± 0.30	0.778 ± 0.30	0.803 ± 0.32	0.759 ± 0.30	0.913 ± 0.17	0.933 ± 0.15	0.330 ± 0.22
	PSPNet	0.744 ± 0.25	0.819 ± 0.24	0.805 ± 0.25	0.801 ± 0.25	0.905 ± 0.17	0.976 ± 0.06	0.366 ± 0.19
	ResNetUNet (R34)	0.738 ± 0.27	0.808 ± 0.26	0.790 ± 0.27	0.782 ± 0.28	**0.914 ± 0.20**	0.976 ± 0.07	0.329 ± 0.18
Data 3, seen seq.	aggcmab	**0.781 ± 0.27**	**0.834 ± 0.28**	0.824 ± 0.28	0.821 ± 0.29	**0.954 ± 0.07**	0.958 ± 0.06	**0.452 ± 0.24**
	AIM_CityU	0.506 ± 0.36	0.587 ± 0.36	0.543 ± 0.37	0.546 ± 0.36	0.877 ± 0.29	0.881 ± 0.13	0.487 ± 0.25
	HoLLYS_ETRI	0.543 ± 0.36	0.623 ± 0.36	0.595 ± 0.36	0.607 ± 0.36	0.908 ± 0.23	0.891 ± 0.12	0.480 ± 0.26
	MLC_SimulaMet	**0.830 ± 0.23**	**0.878 ± 0.23**	**0.866 ± 0.24**	**0.860 ± 0.24**	**0.966 ± 0.05**	**0.975 ± 0.03**	**0.429 ± 0.22**
	sruniga	0.656 ± 0.36	0.714 ± 0.37	0.713 ± 0.37	0.788 ± 0.32	0.783 ± 0.34	0.943 ± 0.07	0.531 ± 0.23
	DeepLabV3+ (R50)	0.746 ± 0.26	0.817 ± 0.24	**0.826 ± 0.24**	0.851 ± 0.25	0.877 ± 0.19	**0.959 ± 0.03**	0.473 ± 0.22
	FCN8	0.625 ± 0.27	0.726 ± 0.27	0.713 ± 0.26	0.736 ± 0.27	0.869 ± 0.23	0.932 ± 0.06	0.528 ± 0.23
	PraNet	0.731 ± 0.30	0.793 ± 0.29	0.821 ± 0.28	0.771 ± 0.27	0.928 ± 0.14	0.927 ± 0.12	0.418 ± 0.20
	PSPNet	0.732 ± 0.27	0.805 ± 0.26	0.818 ± 0.25	**0.853 ± 0.23**	0.852 ± 0.23	0.957 ± 0.03	0.501 ± 0.22
	ResNetUNet (R34)	0.669 ± 0.29	0.751 ± 0.29	0.741 ± 0.29	0.746 ± 0.31	0.916 ± 0.17	0.937 ± 0.06	0.529 ± 0.26
Data 4, unseen seq.	aggcmab	**0.695 ± 0.35**	**0.749 ± 0.35**	**0.729 ± 0.35**	**0.754 ± 0.34**	**0.924 ± 0.21**	**0.970 ± 0.05**	**0.332 ± 0.22**
	AIM_CityU	0.449 ± 0.38	0.516 ± 0.39	0.487 ± 0.39	0.654 ± 0.36	0.701 ± 0.42	0.952 ± 0.06	0.435 ± 0.19
	HoLLYS_ETRI	0.637 ± 0.36	0.700 ± 0.36	0.677 ± 0.35	0.693 ± 0.35	**0.914 ± 0.23**	0.964 ± 0.04	0.391 ± 0.26
	MLC_SimulaMet	**0.684 ± 0.36**	**0.737 ± 0.35**	**0.718 ± 0.36**	0.719 ± 0.36	0.909 ± 0.23	**0.972 ± 0.05**	**0.335 ± 0.22**
	sruniga	0.472 ± 0.39	0.532 ± 0.41	0.509 ± 0.41	**0.752 ± 0.32**	0.648 ± 0.44	0.965 ± 0.05	0.452 ± 0.17
	DeepLabV3+ (R50)	0.613 ± 0.36	0.680 ± 0.36	0.657 ± 0.36	0.718 ± 0.33	0.852 ± 0.29	0.963 ± 0.05	0.381 ± 0.22
	FCN8	0.562 ± 0.37	0.628 ± 0.38	0.597 ± 0.38	0.651 ± 0.36	0.862 ± 0.30	0.960 ± 0.05	0.363 ± 0.23
	PraNet	0.483 ± 0.39	0.543 ± 0.41	0.557 ± 0.41	0.554 ± 0.43	0.858 ± 0.23	0.832 ± 0.20	0.483 ± 0.27
	PSPNet	0.597 ± 0.37	0.662 ± 0.37	0.632 ± 0.38	0.676 ± 0.35	0.872 ± 0.29	0.962 ± 0.05	0.344 ± 0.22
	ResNetUNet (R34)	0.614 ± 0.36	0.678 ± 0.36	0.652 ± 0.37	0.709 ± 0.33	0.878 ± 0.28	0.965 ± 0.04	0.399 ± 0.24

↑: best increasing ↓: best decreasing R34: ResNet34 R50: ResNet50

Supplementary Table 5. Semantic segmentation results for teams ranking below 5th place on out-of-sample data 1, data 2, data 3 and data 4.

Data type	Teams/Method	JC ↑	DSC ↑	F2 ↑	PPV ↑	Recall ↑	ACC ↑	H_d ↓
**Data 1 (NBI-single)**	YCH_THU	0.262 ± 0.29	0.340 ± 0.33	0.383 ± 0.36	0.518 ± 0.41	0.343 ± 0.35	0.877 ± 0.09	0.544 ± 0.18
	Mah_UNM	0.327 ± 0.31	0.413 ± 0.35	0.404 ± 0.36	0.430 ± 0.39	0.696 ± 0.35	0.946 ± 0.06	0.412 ± 0.15
	NDS_MultiUni	0.176 ± 0.26	0.237 ± 0.29	0.259 ± 0.31	0.316 ± 0.38	0.591 ± 0.42	0.912 ± 0.07	0.497 ± 0.17
**Data 2 (WLE-single)**	YCH_THU	0.514 ± 0.34	0.599 ± 0.36	0.640 ± 0.36	0.767 ± 0.32	0.575 ± 0.37	0.934 ± 0.08	0.487 ± 0.20
	Mah_UNM	0.473 ± 0.32	0.569 ± 0.34	0.588 ± 0.35	0.643 ± 0.36	0.652 ± 0.34	0.947 ± 0.08	0.459 ± 0.18
	NDS_MultiUni	0.340 ± 0.28	0.440 ± 0.31	0.459 ± 0.32	0.543 ± 0.36	0.548 ± 0.38	0.918 ± 0.09	0.553 ± 0.20
**Data 3 (seen seq.)**	YCH_THU	0.499 ± 0.32	0.598 ± 0.32	0.649 ± 0.33	0.796 ± 0.32	0.586 ± 0.35	0.893 ± 0.09	0.593 ± 0.16
	Mah_UNM	0.427 ± 0.35	0.509 ± 0.37	0.536 ± 0.39	0.589 ± 0.43	0.713 ± 0.33	0.865 ± 0.13	0.548 ± 0.24
	NDS_MultiUni	0.526 ± 0.32	0.624 ± 0.32	0.674 ± 0.30	0.792 ± 0.29	0.651 ± 0.33	0.915 ± 0.06	0.671 ± 0.19
**Data 4 (unseen seq.)**	YCH_THU	0.328 ± 0.32	0.409 ± 0.36	0.444 ± 0.38	0.747 ± 0.34	0.414 ± 0.37	0.880 ± 0.12	0.880 ± 0.12
	Mah_UNM	0.249 ± 0.32	0.306 ± 0.36	0.302 ± 0.37	0.530 ± 0.43	0.459 ± 0.42	0.928 ± 0.07	0.524 ± 0.19
	NDS_MultiUni	0.249 ± 0.34	0.305 ± 0.35	0.294 ± 0.35	0.429 ± 0.41	0.529 ± 0.42	0.920 ± 0.07	0.920 ± 0.07

↑: best increasing ↓: best decreasing

Supplementary Notes

Related works

Deep learning for detection and localisation of polyps

While frame-based classification methods are used for identifying polyp and non-polyp frames[23–25], detection methods provide both classification and localisation of polyps with in a frame[26,27] which can direct clinicians to the site of interest, and can be additionally used for counting polyps to assess disease burden in patients. With the advancements in object detection architectures, recent methods are end-to-end networks providing better detection performance and improved speed. The state-of-the-art methods are divided into two categories: multi-stage and single-stage. The multi-stage detector methods include Region proposals-Based Convolutional Neural Network (R-CNN)[28], Fast R-CNN[29], Faster R-CNN[30], Region-based fully convolutional networks (R-FCN)[31], Feature Pyramid Network (FPN)[32] and Cascade R-CNN[33]. On the other hand, the One-stage detectors directly provide the predicted output (bounding boxes and object classification) from input images without the region of interest (ROI) proposal stage. The One-stage detector methods include Single-Shot Multibox Detector (SSD)[34], YOLO[35], RetinaNet[21] and Efficientdet[22].

Different studies have been conducted in the literature focusing on polyp detection by employing multi- and single-stage detectors. **Multi-stage Detectors:** Shin et al.[36] used a transfer learning strategy based on Faster R-CNN architecture with the Inception ResNet backbone to detect polyps. Qadir *et al.*[26] adapted Mask R-CNN[37] to detect colorectal polyps and evaluate its performance with different CNN including ResNet50[38], ResNet101[38] and Inception ResNetV2[39] as its feature extractor. Despite the speed limitation, multi-stage detectors are widely used in the detection task of endoscopy data challenges due to their competitive performance on evaluation metrics. **Single-stage Detectors:** Urban *et al.*[27] used YOLO to detect polyps in real-time, resulting in high detection performance. Lee *et al.*[40] employed YOLOv2[41] and validated the proposed approach on four independent datasets. They reported a real-time performance and high sensitivity and specificity on all datasets. Zhang *et al.*[42] proposed the ResYOLO network, adding residual learning modules into the YOLO architecture to train deeper networks. They reported a near-real-time performance for the ResYOLO network depending on the hardware used. Zhang et al.[43] proposed an enhanced SSD named SSD for Gastric Polyps (SSD-GPNet) for real-time gastric polyp detection. SSD-GPNet concatenates feature maps from lower layers and deconvolves higher layers using different pooling techniques. YOLOv3[44] with darknet53 backbone and YOLOv4 showed IOU and average precision (AP) over 0.80% and real-time FPS over 45. Moreover, there exist methods that rely on **anchor-free detectors** to locate the polyps where they claim to detect polyps without the definition of anchors such as CornerNet[45] and ExtremeNet[46]. Zhou *et al.*[47] proposed the CenterNet, which treats each object as a point and increases the speed significantly while ensuring acceptable accuracy. While Wang *et al.*[48] achieved state-of-the-art results on automatic polyp detection in real-time situations using anchor-free object detection methods. In addition to these works, Multi-stage, Single-stage and other types of detectors have been widely used by participants teams in different polyp detection datasets and challenges such as MICCAI'15[49], ROBUST-MIS[50], EAD2019[51] and EndoCV2020[52].

Deep learning for segmentation of polyps

Semantic segmentation is the process of grouping related pixels in an image to an object of the same category. Deep learning has been very successful in the field of the medical domain, convolutional neural networks (CNN) based techniques were suggested to generate complete and precise segmentation outputs without requiring any post-processing. In deep learning, medical segmentation methods can be categorized into four categories: Models based on fully convolutional networks, Models based on Encoder-Decoder architecture, Models based on Pyramid-based architecture and Models based on Dilated Convolution Architecture.

Models based on fully convolutional networks: Brandao et al.[53] proposed three different FCN-based architectures for detection and segmentation of polyps from colonoscopy images. Zhang et al.[54] proposed multi-step practice for the polyp segmentation. The former step includes region proposal generation using FCN, and the latter step uses spatial features and a random forest classifier for the refinement process. A similar method was introduced by Akbari et al.[55] which uses patch selection while training FCN and Otsu thresholding to find the accurate location of polyp. Guo et al.[56] describe two methods based on FCN for Gastrointestinal ImageANALysis (GIANA) polyp segmentation sub-challenge.

Models based on encoder-decoder architecture: Nguyen and Slee[57] proposed multiple deep encoder-decoder networks to capture multi-level contextual information and learn rich features during training. Zhou et al.[58] proposed UNet++, a deeply-supervised encoder-decoder network that showed good results on the polyp segmentation task. Similarly, Jha et. al[59] proposed ResUNet++ that combines series of residual blocks, squeeze and excitation network, atrous spatial pyramid pooling, and attention block. Tomar et al.[60] proposed a dual-decoder attention network (DDANet) that utilizes residual learning and the squeeze and excitation network. Inspired by HRNet[61], Srivastava et al.[62] proposed multi-scale residual fusion network (MSRF-Net) that allows information exchange across multiple scales. Mahmud et al.[63] integrated dilated inception blocks into each unit layer and aggregate the features of the different receptive fields to capture better-generalized feature representations. Huang et al.[64] proposed a low memory traffic, fast and accurate method for the polyp segmentation achieving 86 frames per

second (FPS). Later, Zhang et al.[65] proposed a hybrid method combining both transformer-based network and CNN to capture global dependencies and the low-level spatial features for the segmentation task. Most encoder-decoder architectures were evaluated only on still images. Ji et al.[66] proposed a progressively normalized self-attention network (PNS-Net) for video polyp segmentation.

Models based on pyramid-based architecture: Jia et al.[67] proposed a pyramid-based model named Polyp Net (PLPNet) for automated pixel-level polyp classification in colonoscopy images. Also, Guo et al.[68] employed the Pyramid Scene Parsing Network (PSPNet)[69] with SegNet[70] and U-Net[71] as an ensemble deep learning model. The proposed model achieved a improvement upto 6.38% compared with a single basic trainer.

Models based on dilated convolution architecture: Sun et al.[72] used dilated convolution in the last block of the encoder while Safarov et al.[73] used in all encoder blocks. Though[73] used a mesh of attention blocks and residual block as a decoder, both methods tested there model on CVC-ClinicDB achieving F1-score of 96.106 and 96.043, respectively. Furthermore, nested dilation network (NDN)[74] was designed to segment lesions and tested on the GIANA2018 dataset achieving improvements on Dice upto 3% compared to other methods.

Advantages and limitations of current methods: Methods based on deep learning have attracted considerable interest in the detection and segmentation of polyps in colonoscopy images. The proposed approaches provided high accuracy rates, reducing the risk of missed polyps and enhancing the overall efficacy of colon cancer screening. Limited model generalisability is a critical limiting factor in currently developed methods. While most methods are supervised, the lack of availability of large annotated colonoscopy datasets also becomes another limiting factor for applying polyp detection and segmentation methods, as they tend to be laborious and time-consuming. Additionally, a lack of model interpretability can present difficulties, potentially giving rise to problems in medical settings where understanding is essential.

Method summary of the participants

Below, we summarise the top teams of the EndoCV2021 generalisability assessment challenge for polyp detection and segmentation methods using deep learning. Tabulated summaries are also provided, highlighting the nature of the devised methods and basis of choice in terms of speed and accuracy for detection and segmentation (see Table 3 and Table 4). Methods are detailed in the compiled EndoCV2021 challenge proceeding[75].

Detection Task

- **AIM_CityU:** The team used one-stage anchor-free FCOS[76] as the baseline detection algorithm and adopted ResNeXt-101-DCN with FPN for their final feature extractor. The input images were rescaled to 512×512. For the model optimisation, online (random flipping and multi-scale training) and offline (random rotation, gamma contrast, brightness transformation, etc.) data augmentation strategies were performed to improve the model generalisation. The team minimised cross-entropy loss and used a Stochastic Gradient Descent (SGD) optimiser. The learning rate was set to 0.00261 with the learning rate decay of 0.0005, the NMS threshold was set to 0.01, and the score threshold was set to 0.3..

- **HoLLYS_ETRI:** Standard Mask R-CNN[37] was used with pre-trained weights for the detection and segmentation task. The input images were rescaled to 608×608. An ensemble learning method based on 5-fold cross-validation was used to improve the generalisation performance. For training a single Mask R-CNN, only the data acquired from four centres were used for training and the fifth centre data was used for validation. The final prediction was based on the combination of inference results from five trained models. The polyp localisation for the detection task was done by using the weighted box fusion technique[77] while For the segmentation task, masks from five models were averaged with IoU threshold of 0.6. Data augmentation has been applied to increase data size using RandomBrightness, RandomContrast, RandomSaturation, RandomLighting, RandomCrop, and RandomFlip. The SGD was set as the optimiser to minimise smooth L1-loss with a learning rate of 0.001 and a learning rate decay of 0.0005.

- **JIN_ZJU:** The team used the YOLOV5[78] as the baseline detection algorithm with different data augmentation methods that included hue adjustment, saturation adjustment, value adjustment, rotating, translation, scaling, up-down flipping, left-right flipping, mosaic and mixup. The input images were rescaled to 640×640. BECLogits Loss was employed for the objectness score, while BCEcls loss was for the class probability score. SGD optimisation was chosen with an initial learning rate of 0.01 with a learning rate decay of 0.0005..

- **GECE_VISION:** An ensemble-based polyp detection architecture used the EfficientDet[22] as the base model. The bootstrap aggregating (bagging) technique was utilised to aggregate different versions of the predictors (EfficientDet D0, D1, D2, D3), which were trained on bootstrap replicates of the training set. Data augmentation that included scale jittering, horizontal flipping, and rotations were used to increase the variance and improve the model's generalisation capability. The Adam optimizer was used to minimise focal loss. Learning rate scheduling was implemented, reducing

the learning rate by a factor of 0.2 from 0.0001 whenever the validation set loss did not decrease over the previous 10 epochs.

Segmentation Task

- **aggcmab:** The team improved their previously developed cascaded double encoder-decoder convolutional neural network[79] by increasing the encoder representation capability and adapting to a multi-site sampling technique. The first encoder-decoder generated an initial attempt to segment the polyp by extracting features and downsampling spatial resolutions while increasing the number of channels by learning convolutional filters. The output from the first network acted as an input for the second encoder-decoder along with the original image. A binary cross-entropy (BCE) loss was minimized using the SGD optimiser with a learning rate of 0.01 with rate decay of $1e^{-8}$ every 25 epochs. The training images were resized to 640×512 pixels, and data augmentation, including random rotations, vertical and horizontal flipping, contrast, saturation and brightness changes, was applied.

- **AIM_CityU:** The team adopted HRNet[61] as the backbone to maintain the high-resolution representations in a multi-scale feature fusion mechanism. The team proposed a low-rank module to distribute feature maps in the high dimensional space to a low dimensional manifold to eliminate noisy information in segmentation predictions and enhance model generalisation. The training images were resized to 256×256 pixels, and various data augmentation strategies, including random flipping, rotation, colour shift (brightness, colour, sharpness, and contrast) and Gaussian noise, were performed to improve the model generalisation further. BCE and dice loss (DSC) were utilized to optimise the model. The SGD optimiser used with a learning rate of 0.01, the momentum of 0.9, and the weight decay of 0.0005.

- **HoLLYS_ETRI:** The team used the same method for the detection task discussed previously.

- **MLC_SimulaMet:** Two ensemble models using well-known segmentation models; namely UNet++[58], FPN[32], DeepLabv3[80], DeepLabv3+[81] and novel TriUNet for their DivergentNet ensemble model. The TriUNet ensemble model used three UNet[71] architectures in an ensemble fashion. Here, the TriUNet model took a single image as input, which was passed through two separate UNet models with different randomized weights. The output of both models was then concatenated before being passed through a third UNet model to predict the final segmentation mask. The whole TriUNet network was trained as a single unit. The input images were resized to 256×256 with several data augmentation methods applied to increase data size, such as horizontal flip, shift scale rotation, resizing, additive Gaussian noise, perspective shift, contrast limited adaptive histogram equalization (CLAHE), random brightness, random gamma, random sharpen, random blur, random motion blur, random contrast, and hue saturation. The learning rate was set to 0.0001 and reduced to 0.00001 after 50 epochs using Adam optimiser to minimise BCE and DSE loss functions.

- **sruniga:** A lightweight deep learning-based algorithm was used to meet the real-time clinical need. The proposed network applied the HarDNet-MSEG[64] as the backbone network with reduced shortcuts. Moreover, a proposed data augmentation strategy for realising an improved generalisable model was used. For training the model, the dataset was split into 80% training and 20% validation and images were resized to 352×352 pixels. They used an Adam optimiser to minimise BCE loss with a learning rate of $1e^{-5}$ for all experiments.

- **Mah_UNM:** The team proposed a modified SegNet[70] by embedding Gated recurrent units (GRU) units[82] within the convolution layers for the improved segmentation of polyps. The hyperparameters were set to the original SegNet with a learning rate of 0.005 and batch size of 4. The provided dataset was split into 80% training and 20% validation, and the weighted cross-entropy loss was optimized using an Adam optimizer. Data augmentation has not been utilized.

- **NDS_MultiUni:** A cascaded ensemble model made of four different MultiResUNet[83] architectures with each model generating an output mask. Afterwards, the four predicted outputs were averaged together to produce the final segmentation mask. Each model was trained for 100 epochs with the same hyper-parameter setting. The input images were resized to 256×256 with no data augmentation, and training was done with a batch size of 8. A binary cross-entropy was used as a loss function optimized using an Adam optimizer with a learning rate 0.001.

- **YCH_THU:** The team used an existing parallel reverse attention network referred to as "PraNet"[84]. They extracted multi-level features from colonoscopy images utilizing a parallel res2Net-based network. Moreover, the segmentation results were post-processed to remove uncertain pixels and enhance polyp boundaries. The images were resized to 512×512 pixels, and the dataset was split into 80% training and 20% validation. The model was trained for 300 epochs with batch size 20, learning rate of 0.0001 with learning rate decay of 0.1 and using Adam optimizer. No data augmentation has been applied.

References

1. Bernal, J., Sánchez, J. & Vilarino, F. Towards automatic polyp detection with a polyp appearance model. *Pattern Recognit.* **45**, 3166–3182 (2012).
2. Bernal, J. *et al.* Wm-dova maps for accurate polyp highlighting in colonoscopy: Validation vs. saliency maps from physicians. *Comput. Med. Imaging Graph.* **43**, 99–111 (2015).
3. Bernal, J. & Aymeric, H. Miccai endoscopic vision challenge polyp detection and segmentation. *Web-page 2017 Endosc. Vis. Chall.* (2017).
4. Ali, S. *et al.* Endoscopy disease detection challenge 2020. *arXiv preprint arXiv:2003.03376* (2020).
5. Ali, S. *et al.* Deep learning for detection and segmentation of artefact and disease instances in gastrointestinal endoscopy. *Med. Image Analysis* **70**, 102002, DOI: 10.1016/j.media.2021.102002 (2021).
6. Silva, J., Histace, A., Romain, O., Dray, X. & Granado, B. Toward embedded detection of polyps in wce images for early diagnosis of colorectal cancer. *Int. J. Comput. Assist. Radiol. Surg.* **9**, 283–293 (2014).
7. Tajbakhsh, N., Gurudu, S. R. & Liang, J. Automated polyp detection in colonoscopy videos using shape and context information. *IEEE transactions on medical imaging* **35**, 630–644 (2015).
8. Borgli, H. *et al.* Hyperkvasir, a comprehensive multi-class image and video dataset for gastrointestinal endoscopy. *Sci. Data* **7**, 1–14 (2020).
9. Jha, D. *et al.* Kvasir-SEG: A segmented polyp dataset. In *International Conference on Multimedia Modeling*, 451–462 (2020).
10. Jha, D. *et al.* Gastrovision: A multi-class endoscopy image dataset for computer aided gastrointestinal disease detection. *arXiv preprint arXiv:2307.08140* (2023).
11. Misawa, M. *et al.* Development of a computer-aided detection system for colonoscopy and a publicly accessible large colonoscopy video database (with video). *Gastrointest. endoscopy* **93**, 960–967 (2021).
12. Azagra, P. *et al.* Endomapper dataset of complete calibrated endoscopy procedures. *Sci. Data* **10**, 671 (2023).
13. Sánchez-Peralta, L. F. *et al.* Piccolo white-light and narrow-band imaging colonoscopic dataset: A performance comparative of models and datasets. *Appl. Sci.* **10**, 8501 (2020).
14. Wang, G. Replication data for: Colonoscopy polyp detection and classification: Dataset creation and comparative evaluations. *Harv. Dataverse* (2021).
15. Ali, S. *et al.* PolypGen: a multi-center polyp detection and segmentation dataset for generalisability assessment. *arXiv preprint arXiv:2106.04463* (2021).
16. Wuyang, L. *et al.* Joint polyp detection and segmentation with heterogeneous endoscopic data. In *Proceedings of the 3rd International Workshop and Challenge on Computer Vision in Endoscopy (EndoCV 2021) co-located with with the 18th IEEEInternational Symposium on Biomedical Imaging (ISBI 2021), Nice, France, April 13, 2021*, vol. 2886, 69–79 (CEUR-WS.org, 2021).
17. Polat, G., Isik-Polat, E., Kayabay, K. & Temizel, A. Polyp detection in colonoscopy images using deep learning and bootstrap aggregation. In *Proceedings of the 3rd International Workshop and Challenge on Computer Vision in Endoscopy (EndoCV 2021) co-located with with the 18th IEEEInternational Symposium on Biomedical Imaging (ISBI 2021), Nice, France, April 13, 2021*, vol. 2886, 90–100 (CEUR-WS.org, 2021).
18. Honga, A., Leeb, G., Leec, H., Seod, J. & Yeoe, D. Deep learning model generalization with ensemble in endoscopic images. In *Proceedings of the 3rd International Workshop and Challenge on Computer Vision in Endoscopy (EndoCV 2021) co-located with with the 18th IEEEInternational Symposium on Biomedical Imaging (ISBI 2021), Nice, France, April 13, 2021*, vol. 2886, 80–89 (2021).
19. Gana, T., Zhaa, Z., Hua, C. & Jina, Z. Detection of polyps during colonoscopy procedure using yolov5 network. In *Proceedings of the 3rd International Workshop and Challenge on Computer Vision in Endoscopy (EndoCV 2021) co-located with with the 18th IEEEInternational Symposium on Biomedical Imaging (ISBI 2021), Nice, France, April 13, 2021*, vol. 2886, 101–110 (CEUR-WS.org, 2021).
20. Bochkovskiy, A., Wang, C. & Liao, H. M. Yolov4: Optimal speed and accuracy of object detection. *CoRR* **abs/2004.10934** (2020). 2004.10934.
21. Lin, T.-Y., Goyal, P., Girshick, R., He, K. & Dollár, P. Focal loss for dense object detection. In *Proceedings of the IEEE international conference on computer vision*, 2980–2988 (2017).

22. Tan, M., Pang, R. & Le, Q. V. Efficientdet: Scalable and efficient object detection. In *Proceedings of the IEEE/CVF conference on computer vision and pattern recognition*, 10781–10790 (2020).

23. Tajbakhsh, N., Gurudu, S. R. & Liang, J. Automatic polyp detection in colonoscopy videos using an ensemble of convolutional neural networks. In *2015 IEEE 12th International Symposium on Biomedical Imaging (ISBI)*, 79–83 (IEEE, 2015).

24. Park, S. Y. & Sargent, D. Colonoscopic polyp detection using convolutional neural networks. In *Medical Imaging 2016: Computer-Aided Diagnosis*, vol. 9785, 978528 (International Society for Optics and Photonics, 2016).

25. Ribeiro, E., Uhl, A. & Häfner, M. Colonic polyp classification with convolutional neural networks. In *2016 IEEE 29th International Symposium on Computer-Based Medical Systems (CBMS)*, 253–258 (IEEE, 2016).

26. Qadir, H. A. *et al.* Polyp detection and segmentation using mask r-cnn: Does a deeper feature extractor cnn always perform better? In *2019 13th International Symposium on Medical Information and Communication Technology (ISMICT)*, 1–6 (2019).

27. Urban, G. *et al.* Deep learning localizes and identifies polyps in real time with 96% accuracy in screening colonoscopy. *Gastroenterology* **155**, 1069–1078 (2018).

28. Girshick, R., Donahue, J., Darrell, T. & Malik, J. Rich feature hierarchies for accurate object detection and semantic segmentation. In *Proceedings of the IEEE conference on computer vision and pattern recognition*, 580–587 (2014).

29. Girshick, R. Fast r-cnn. In *Proceedings of the IEEE international conference on computer vision*, 1440–1448 (2015).

30. Ren, S., He, K., Girshick, R. & Sun, J. Faster r-cnn: Towards real-time object detection with region proposal networks. In *Advances in neural information processing systems*, 91–99 (2015).

31. Dai, J., Li, Y., He, K. & Sun, J. R-FCN: Object detection via region-based fully convolutional networks. In *Advances in neural information processing systems*, 379–387 (2016).

32. Lin, T.-Y. *et al.* Feature pyramid networks for object detection. In *Proceedings of the IEEE conference on computer vision and pattern recognition*, 2117–2125 (2017).

33. Cai, Z. & Vasconcelos, N. Cascade r-cnn: Delving into high quality object detection. In *Proceedings of the IEEE Conference on Computer Vision and Pattern Recognition*, 6154–6162 (2018).

34. Liu, W. *et al.* Ssd: Single shot multibox detector. In *European conference on computer vision*, 21–37 (Springer, 2016).

35. Redmon, J., Divvala, S., Girshick, R. & Farhadi, A. You only look once: Unified, real-time object detection. In *Proceedings of the IEEE conference on computer vision and pattern recognition*, 779–788 (2016).

36. Y., S., H. A., Q., L., A., J., B. & I., B. Automatic colon polyp detection using region based deep cnn and post learning approaches. *IEEE Access* **6**, 40950–40962 (2018).

37. He, K., Gkioxari, G., Dollár, P. & Girshick, R. Mask r-cnn. In *Proceedings of the IEEE international conference on computer vision*, 2961–2969 (2017).

38. He, K., Zhang, X., Ren, S. & Sun, J. Deep residual learning for image recognition. In *Proceedings of the IEEE conference on computer vision and pattern recognition*, 770–778 (2016).

39. Szegedy, C., Ioffe, S., Vanhoucke, V. & Alemi, A. A. Inception-v4, inception-resnet and the impact of residual connections on learning. In *Thirty-first AAAI conference on artificial intelligence* (2017).

40. Lee, J. Y. *et al.* Real-time detection of colon polyps during colonoscopy using deep learning: systematic validation with four independent datasets. *Sci. reports* **10**, 1–9 (2020).

41. Redmon, J. & Farhadi, A. Yolo9000: better, faster, stronger. In *Proceedings of the IEEE conference on computer vision and pattern recognition*, 7263–7271 (2017).

42. Zhang, R., Zheng, Y., Poon, C. C., Shen, D. & Lau, J. Y. Polyp detection during colonoscopy using a regression-based convolutional neural network with a tracker. *Pattern recognition* **83**, 209–219 (2018).

43. Zhang, X. *et al.* Real-time gastric polyp detection using convolutional neural networks. *PloS one* **14**, e0214133 (2019).

44. Farhadi, A. & Redmon, J. Yolov3: An incremental improvement. In *Computer Vision and Pattern Recognition*, 1804–2767 (Springer Berlin/Heidelberg, Germany, 2018).

45. Law, H. & Deng, J. Cornernet: Detecting objects as paired keypoints. In *Proceedings of the European Conference on Computer Vision (ECCV)*, 734–750 (2018).

46. Zhou, X., Zhuo, J. & Krahenbuhl, P. Bottom-up object detection by grouping extreme and center points. In *Proceedings of the IEEE Conference on Computer Vision and Pattern Recognition*, 850–859 (2019).

47. Zhou, X., Wang, D. & Krähenbühl, P. Objects as points. *arXiv preprint arXiv:1904.07850* (2019).

48. Wang, D. *et al.* AFP-Net: Realtime Anchor-Free Polyp Detection in Colonoscopy. In *2019 IEEE 31st International Conference on Tools with Artificial Intelligence (ICTAI)*, 636–643 (IEEE, 2019).

49. Bernal, J. *et al.* Comparative validation of polyp detection methods in video colonoscopy: Results from the miccai 2015 endoscopic vision challenge. *IEEE Transactions on Med. Imaging* **36**, 1231–1249, DOI: 10.1109/TMI.2017.2664042 (2017).

50. Ross, T. *et al.* Robust medical instrument segmentation challenge 2019. *arXiv preprint arXiv:2003.10299* (2020).

51. Ali, S. *et al.* Endoscopy Artefact Detection (EAD) dataset, DOI: https://doi.org/10.17632/c7fjbxcgj9.1 (2019).

52. Ali, S. *et al.* Deep learning for detection and segmentation of artefact and disease instances in gastrointestinal endoscopy. *Med. Image Analysis* **70**, 102002 (2021).

53. Brandao, P. *et al.* Fully convolutional neural networks for polyp segmentation in colonoscopy. In *Medical Imaging 2017: Computer-Aided Diagnosis*, vol. 10134, 101340F (International Society for Optics and Photonics, 2017).

54. Zhang, L., Dolwani, S. & Ye, X. Automated polyp segmentation in colonoscopy frames using fully convolutional neural network and textons. In *Annual Conference on Medical Image Understanding and Analysis*, 707–717 (Springer, 2017).

55. Akbari, M. *et al.* Polyp segmentation in colonoscopy images using fully convolutional network. In *2018 40th Annual International Conference of the IEEE Engineering in Medicine and Biology Society (EMBC)*, 69–72 (IEEE, 2018).

56. Guo, Y. B. & Matuszewski, B. Giana polyp segmentation with fully convolutional dilation neural networks. In *Proceedings of the 14th International Joint Conference on Computer Vision, Imaging and Computer Graphics Theory and Applications*, 632–641 (SCITEPRESS-Science and Technology Publications, 2019).

57. Nguyen, N.-Q. & Lee, S.-W. Robust boundary segmentation in medical images using a consecutive deep encoder-decoder network. *Ieee Access* **7**, 33795–33808 (2019).

58. Zhou, Z., Siddiquee, M. M. R., Tajbakhsh, N. & Liang, J. UNet++: redesigning skip connections to exploit multiscale features in image segmentation. *IEEE Transactions on Med. Imaging* **39**, 1856–1867 (2019).

59. Jha, D. *et al.* A comprehensive analysis of classification methods in gastrointestinal endoscopy imaging. *Med. image analysis* **70**, 102007 (2021).

60. Tomar, N. K. *et al.* Ddanet: Dual decoder attention network for automatic polyp segmentation. In *International Conference on Pattern Recognition*, 307–314 (2021).

61. Wang, J. *et al.* Deep high-resolution representation learning for visual recognition. *IEEE transactions on pattern analysis machine intelligence* (2020).

62. Srivastava, A. *et al.* MSRF-Net: A Multi-Scale Residual Fusion Network for Biomedical Image Segmentation. *IEEE J. Biomed. Heal. informatics* (2021).

63. Mahmud, T., Paul, B. & Fattah, S. A. PolypSegNet: a modified encoder-decoder architecture for automated polyp segmentation from colonoscopy images. *Comput. Biol. Medicine* **128**, 104119 (2021).

64. Huang, C.-H., Wu, H.-Y. & Lin, Y.-L. HarDNet-MSEG: A Simple Encoder-Decoder Polyp Segmentation Neural Network that Achieves over 0.9 Mean Dice and 86 FPS. *arXiv preprint arXiv:2101.07172* (2021).

65. Zhang, Y., Liu, H. & Hu, Q. Transfuse: Fusing transformers and CNNs for medical image segmentation. *arXiv preprint arXiv:2102.08005* (2021).

66. Ji, G.-P. *et al.* Progressively normalized self-attention network for video polyp segmentation. *arXiv preprint arXiv:2105.08468* (2021).

67. Jia, X. *et al.* Automatic Polyp Recognition in Colonoscopy Images Using Deep Learning and Two-Stage Pyramidal Feature Prediction. *IEEE Transactions on Autom. Sci. Eng.* **17** (2020).

68. Guo, X. *et al.* Automated polyp segmentation for colonoscopy images: A method based on convolutional neural networks and ensemble learning. *Med. Phys.* **46**, 5666–5676 (2019).

69. Zhao, H., Shi, J., Qi, X., Wang, X. & Jia, J. Pyramid scene parsing network. *Proc. - 30th IEEE Conf. on Comput. Vis. Pattern Recognition, CVPR 2017* DOI: 10.1109/CVPR.2017.660 (2017).

70. Badrinarayanan, V., Kendall, A. & Cipolla, R. Segnet: A deep convolutional encoder-decoder architecture for image segmentation. *IEEE transactions on pattern analysis machine intelligence* **39**, 2481–2495 (2017).

71. Ronneberger, O., Fischer, P. & Brox, T. U-net: Convolutional networks for biomedical image segmentation. In *International Conference on Medical image computing and computer-assisted intervention*, 234–241 (Springer, 2015).

72. Sun, X., Zhang, P., Wang, D., Cao, Y. & Liu, B. Colorectal polyp segmentation by u-net with dilation convolution. In *2019 18th IEEE International Conference On Machine Learning And Applications (ICMLA)*, 851–858 (IEEE, 2019).

73. Safarov, S. & Whangbo, T. K. A-DenseUNet: adaptive densely connected unet for polyp segmentation in colonoscopy images with atrous convolution. *Sensors* **21**, 1441 (2021).

74. Wang, L. *et al.* Nested Dilation Network (NDN) for Multi-Task Medical Image Segmentation. *IEEE Access* **7**, 44676–44685, DOI: 10.1109/ACCESS.2019.2908386 (2019).

75. Ali, S., Ghatwary, N. M., Jha, D. & Halvorsen, P. (eds.). *Proceedings of the 3rd International Workshop and Challenge on Computer Vision in Endoscopy (EndoCV 2021) co-located with with the 18th IEEE International Symposium on Biomedical Imaging (ISBI 2021), Nice, France, April 13, 2021*, vol. 2886 of *CEUR Workshop Proceedings* (CEUR-WS.org, 2021).

76. Tian, Z., Shen, C., Chen, H. & He, T. Fcos: Fully convolutional one-stage object detection. In *Proceedings of the IEEE/CVF international conference on computer vision*, 9627–9636 (2019).

77. Solovyev, R., Wang, W. & Gabruseva, T. Weighted boxes fusion: Ensembling boxes from different object detection models. *Image Vis. Comput.* **107**, 104117, DOI: https://doi.org/10.1016/j.imavis.2021.104117 (2021).

78. Jocher, G. YoloV5. https://github.com/ultralytics/yolov5 (2021).

79. Galdran, A., Carneiro, G. & Ballester, M. A. G. Double encoder-decoder networks for gastrointestinal polyp segmentation. In *Pattern Recognition. ICPR International Workshops and Challenges: Virtual Event, January 10–15, 2021, Proceedings, Part I*, 293–307 (Springer International Publishing, 2021).

80. Chen, L.-C., Papandreou, G., Schroff, F. & Adam, H. Rethinking atrous convolution for semantic image segmentation. *arXiv preprint arXiv:1706.05587* (2017).

81. Chen, L.-C., Zhu, Y., Papandreou, G., Schroff, F. & Adam, H. Encoder-decoder with atrous separable convolution for semantic image segmentation. In *Proceedings of the European conference on computer vision (ECCV)*, 801–818 (2018).

82. Cho, K. *et al.* Learning phrase representations using rnn encoder-decoder for statistical machine translation. *arXiv preprint arXiv:1406.1078* (2014).

83. Ibtehaz, N. & Rahman, M. S. MultiResUNet: Rethinking the U-Net architecture for multimodal biomedical image segmentation. *Neural Networks* **121**, 74–87 (2020).

84. Fan, D.-P. *et al.* Pranet: Parallel reverse attention network for polyp segmentation. In *International Conference on Medical Image Computing and Computer-Assisted Intervention*, 263–273 (Springer, 2020).
